# Metabolic engineering of *Acremonium chrysogenum* for improving cephalosporin C production independent of methionine stimulation

**DOI:** 10.1186/s12934-018-0936-5

**Published:** 2018-06-07

**Authors:** Jiajia Liu, Wenyan Gao, Yuanyuan Pan, Gang Liu

**Affiliations:** 1 State Key Laboratory of Mycology, Institute of Microbiology, Chinese Academy of Sciences, Beijing, 100101 China; 2 University of Chinese Academy of Sciences, Beijing, 100049 China

**Keywords:** *Acremonium chrysogenum*, *Acppm1*, *AcsamS*, Cephalosporin C (CPC), *mecB*, Metabolic engineering, Methionine stimulation, *S*-Adenosylmethionine (SAM)

## Abstract

**Background:**

Cephalosporin C (CPC) produced by *Acremonium chrysogenum* is one of the most important drugs for treatment of bacterial infectious diseases. As the major stimulant, methionine is widely used in the industrial production of CPC. In this study, we found methionine stimulated CPC production through enhancing the accumulation of endogenous *S*-adenosylmethionine (SAM). To overcome the methionine dependent stimulation of CPC production, the methionine cycle of *A. chrysogenum* was reconstructed by metabolic engineering.

**Results:**

Three engineered strains were obtained by overexpressing the SAM synthetase gene *AcsamS* and the cystathionine-γ-lyase gene *mecB*, and disrupting a SAM dependent methyltransferase gene *Acppm1*, respectively. Overexpression of *AcsamS* resulted in fourfold increase of CPC production which reached to 129.7 µg/mL. Disruption of *Acppm1* also increased CPC production (up to 135.5 µg/mL) through enhancing the accumulation of intracellular SAM. Finally, an optimum recombinant strain (Acppm1DM-mecBOE) was constructed through overexpressing *mecB* in the *Acppm1* disruption mutant. In this strain, CPC production reached to the maximum value (142.7 µg/mL) which was 5.5-fold of the wild-type level and its improvement was totally independent of methionine stimulation.

**Conclusions:**

In this study, we constructed a recombinant strain in which the improvement of CPC production was totally independent of methionine stimulation. This work provides an economic route for improving CPC production in *A. chrysogenum* through metabolic engineering.

**Electronic supplementary material:**

The online version of this article (10.1186/s12934-018-0936-5) contains supplementary material, which is available to authorized users.

## Background

Cephalosporin C (CPC), as a major β-lactam antibiotic, has been widely used for treatment of bacterial infectious diseases [1]. The cephalosporin biosynthetic gene cluster has been identified and well studied in its producing strain *Acremonium chrysogenum* [2–4]. Besides the regulation of CefR, CPC production was also influenced by other factors, especially the stimulation of exogenous methionine [5]. Previous studies indicated four possible reasons for the methionine stimulation in CPC production: (1) methionine is used as the sulfur precursor of CPC [6]; (2) methionine stimulates expression of the cephalosporin biosynthetic genes [7, 8]; (3) methionine improves the activity of cystathionine-γ-lyase (MecB) in the reverse transsulfuration pathway [9]; (4) methionine has an effect on mycelia morphological differentiation which is related with CPC production [10, 11]. Although the related debate has existed for decades, the main reason for methionine stimulation on CPC production still remains elusive.

**Fig. 1:**
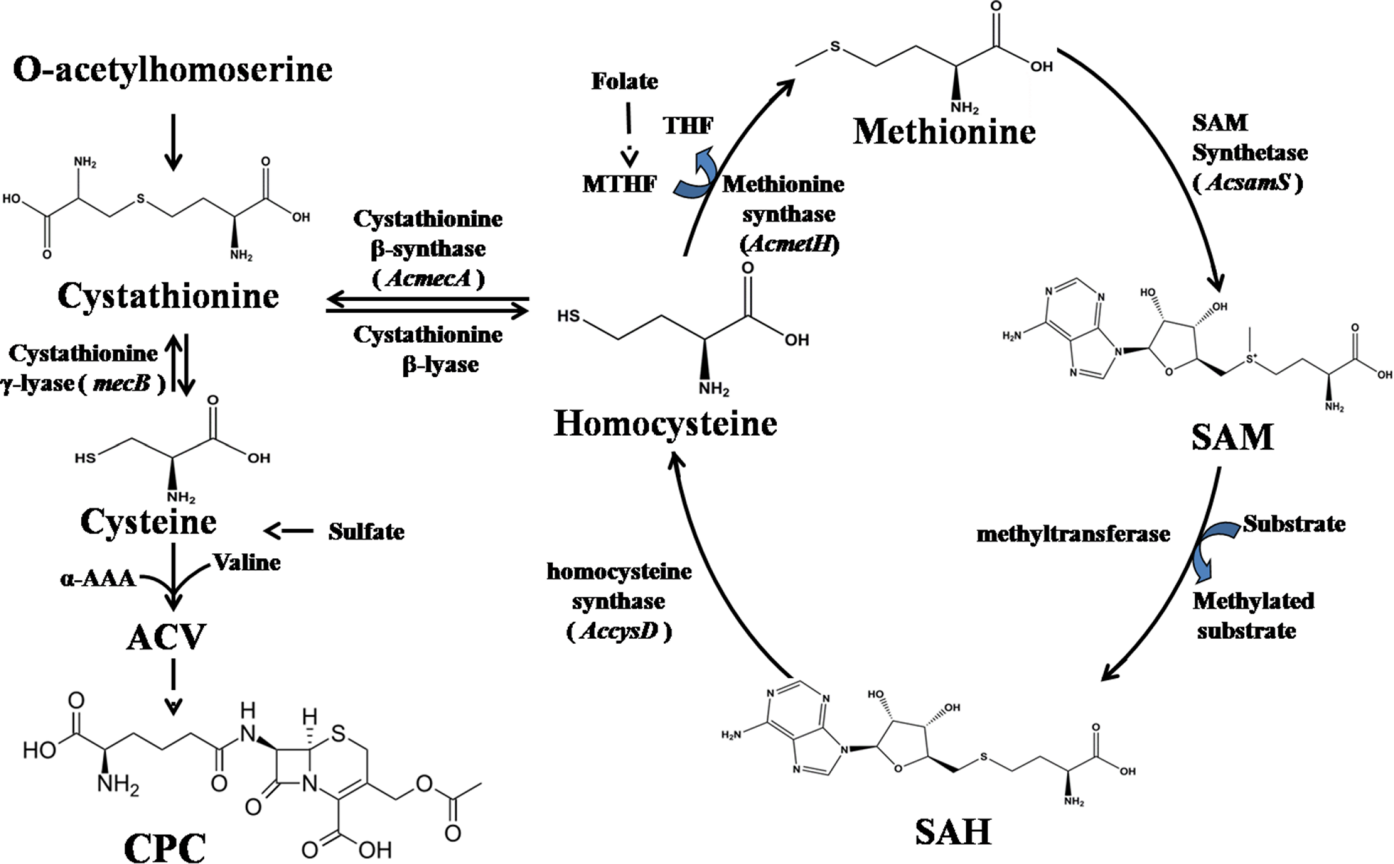
Methionine cycle and the biosynthetic pathway of CPC in *Acremonium chrysogenum.* MTHF, methyltetrahydrofolate; THF, tetrahydrofolate; SAM, *S*-adenosylmethionine; SAH, *S*-adenosylhomocysteine; AC, the tripeptide δ-(l-a-aminoadipyl)-l-cysteinyl-d-valine which is catalyzed by ACV synthetase encoded by *pcbAB*; CPC, cephalosporin C; α-AAA, α-aminoadipic acid

Methionine, a sulfur-containing essential amino acid, participates in complex and crucial metabolism in vivo. In methionine cycle, methionine is adenylated to form *S*-adenosylmethionine (SAM) by SAM synthetase. Subsequently SAM donates its methyl group to methyl acceptors via numerous methyltransferases, forming methylated products and *S*-adenosylhomocysteine (SAH). Then, SAH is cleaved and generates homocysteine. Homocysteine, locating at an important metabolic branch point, can be removed sulfur to generate l-cysteine through transsulfuration pathway. l-cysteine is one of the precursors of CPC in *A. chrysogenum*. On the other hand, homocysteine can be remethylated to form methionine via methionine synthetase (Fig. 1). Through methionine cycle, the sulfur atom is provided for l-cysteine formation through a reverse transsulfuration pathway [12]. Based on methionine cycle, the formation of l-cysteine could be increased through enhancing the intracellular SAM concentration. Therefore, it could be one of the reasons for methionine stimulation on CPC production. Recently, it was reported that the autotrophic sulfur assimilation is important for CPC production in *A. chrysogenum* A3/2 strain [13]. Since two biosynthetic routes for sulfur assimilation exist, different strains of *A. chrysogenum* could use different pathway and it could result in disparity of methionine stimulation on CPC production in different strains. SAM is also involved in many biochemical processes, especially the transmethylation in which SAM serves as the principal biological methyl donor. SAM is synthesized by SAM synthetase using methionine and ATP as substrates [14]. It has been reported that almost half of the daily intake of methionine is converted to SAM in liver [15]. Abnormalities in SAM metabolism have been well studied in liver diseases, injury and cancer [16]. However, the variation of SAM levels did not affect the growth rate and morphogenesis in *Neurospora crassa* [17]. While, addition of exogenous SAM in a concentration from 2 µM to 1 mM led to a significant increase of the antibiotic production in *Streptomyces coelicolor* M145 [18]. In *Bacillus subtilis*, accumulation of SAM also caused antibiotic overproduction [19]. Although SAM is the methyl donor in all known biological methylation reactions of animals or humans [15], it is absolutely ignored in *A. chrysogenum*.

Here we found the endogenous concentration of SAM was significantly increased during fermentation of *A. chrysogenum* when the MDFA medium was supplemented with methionine. As a consequence, we hypothesized that the methionine stimulation on CPC production is due to the accumulation of endogenous SAM. Therefore, we constructed three recombinant strains which accumulated intracellular SAM in two ways: enhancing the SAM synthesis (“open source”) and blocking the SAM consumption (“throttling control”). Finally, an optimum engineered strain which produced CPC totally independent of methionine stimulation was constructed and the CPC production in this strain increased 5.5-fold compared with the wild-type. To our knowledge, this is the first report of an engineered strain in which improvement of CPC production totally independent of methionine stimulation. This work provides an economic route for improving cephalosporin C production of *A. chrysogenum* through metabolic engineering.

## Methods

### Strains, plasmids, media and growth conditions

Strains and plasmids used in this study were listed in Additional file 1: Table S1. For conidiation, *A. chrysogenum* and its derivatives were grown in LPE medium (per liter, 1.0 g glucose, 2.0 g yeast extraction, 1.5 g NaCl, 10 g CaCl_2_, 25.0 g agar, pH 6.8). For fungal growth and total DNA extraction, the TSA medium (per liter, 17.0 g tryptone, 3.0 g soy peptone, 2.5 g glucose, 5.0 g NaCl, 2.5 g K_2_HPO_4_∙3H_2_O, 15.0 g agar, pH 7.0) was used. To screen the constructed strains, the TSA medium with 50 μg/mL hygromycin B, 10 μg/mL bleomycin or 200 μg/mL G418 was used. Minimal medium (MM), induction medium (IM) and co-cultivation medium (CM) were used for *Agrobacterium tumefaciens*-mediated transformation (ATMT) of *A. chrysogenum* [20]. For fermentation, the modified MDFA medium with or without methionine was used as described previously [20]. The *Escherichia coli* strains were grown at 37 °C in Luria–Bertani (LB) medium (per liter, 10.0 g tryptone, 5.0 g yeast extraction, 10.0 g NaCl, pH 7.0) supplemented with necessary antibiotics for propagating plasmids. *Bacillus subtilis* CGMCC 1.1630 was used for detection of CPC production.

### Cloning and sequencing analysis of *AcsamS*, *Acppm1* and *mecB*

All primers used in this study were listed in Additional file 1: Table S2. Using the BLASTX program, the whole genomic DNA sequence of *A. chrysogenum* CGMCC 3.3795 (unpublished) was searched in the non-redundant protein database of National Center for Biotechnology Information (NCBI). Two query sequences were identified as the SAM synthetase gene (named *AcsamS*, GenBank Accession No. MG356328), the cystathionine-γ-lyase gene (*mecB*, GenBank Accession No. MG356327), respectively. In addition, 23 query sequences encoding the putative SAM dependent methyltransferases were found, and one of them was identified by transcriptional analysis as the SAM consumption major gene (named *Acppm1*, GenBank Accession No. MG356326). For transcriptional analysis, the total RNA was isolated from the *A. chrysogenum* wild-type strain (WT) and its derivatives grown for 5 days in the modified MDFA medium with addition of extra 500 μM SAM.

For cloning *AcsamS*, *Acppm1* and *mecB*, WT was grown in TSA liquid medium at 28 °C and the mycelia were harvested after 48 h incubation. After dried with filter paper, the mycelia were ground in liquid nitrogen using sterilized mortar and pestle. The genomic DNA and the total RNA were isolated with DNA Quick Plant System (TianGen, China) and Trizol Reagent (Invitrogen, USA) according to the commercial manual, respectively. The total RNA was treated with RQ1 RNase (free DNase) (Promega, USA) to remove the genomic DNA. The cDNA of *A. chrysogenum* was obtained by reverse transcription of the total RNA using the PrimeScript™ RT reagent kit (TaKaRa, Japan). The DNA and cDNA of *AcsamS*, *Acppm1* and *mecB* were amplified with primers AcsamsOE-F/R, Acppm1OE-F/R and mecB-F/R, respectively. Then, these amplified DNA fragments were cloned into the vector pEASY-Blunt (TransGen, China). Finally, they were verified by sequencing (GENEWIZ, China). Reverse transcription PCR (RT-PCR) and real-time RT-PCR were performed as described previously [21, 22].

### Strain constructions

Using the *A. chrysogenum* genomic DNA as template, two DNA fragments containing the putative promoter and terminator regions of *Acgapdh* (GenBank Accession No. MF383617) were amplified with primers Pgpd-F/R and Tgpd-F/R, and named Pgpd and Tgpd, respectively. Then Pgpd and Tgpd were cloned into pEASY-Blunt to generate pEBPgpd and pEBTgpd. After verified by sequencing, pEBPgpd was digested with *Sal*I and pEBTgpd was digested with *Spe*I, the resulting DNA fragments containing Pgpd and Tgpd were ligated into the corresponding sites of pAgHG to generate pAg1PT-G418. The resulting plasmid pAg1PT-G418 was introduced into WT through ATMT as described previously [20]. The G418 resistant transformants were selected on the TSA medium with 250 μg/mL G418 and 400 μg/mL cefotaxime and one of them was chosen randomly as control in subsequent experiments. To overexpress *AcsamS*, the entire *AcsamS* was amplified from the cDNA of *A. chrysogenum* with primers AcsmasOE-F/R and cloned into pEASY-Blunt to give pEBAcsamsOE. After verified by sequencing, pEBAcsamsOE was digested with *Asc*I/*Pac*I and the fragment containing *AcsamS* was cloned into the corresponding site of pAg1PT-G418. The resulting plasmid pAg1PT-G418::AcsamS was finally introduced into WT through ATMT. The G418 resistant transformants were selected on the TSA medium with 250 μg/mL G418 and 400 μg/mL cefotaxime. The *AcsamS* overexpressed strain (AcsamsOE) was identified by PCR with primers G418-F/R, and real-time RT-PCR with primers RT-Acsams-F/R.

To construct the *Acppm1* disruption mutant (Acppm1DM), a 2031 bp DNA fragment corresponding to the upstream of *Acppm1* (extending from positions − 2198 to − 167 with respect to the *Acppm1* translation start point) and a 2245 bp DNA fragment corresponding to the downstream of *Acppm1* (extending from positions + 1300 to + 3545 with respect to the *Acppm1* translation start point) were amplified from WT with primers Acppm1LB-F/R and Acppm1RB-F/R, respectively. Then these two DNA fragments were cloned into pEASY-Blunt and give pEBppm1LB and pEBppm1RB. After verified by sequencing, pEBppm1LB was digested with *Apa*I. The digested DNA fragment containing the *Acppm1* upstream sequence was subcloned into the corresponding sites of pAgHB to generate pAgHBppm1LB. Then pEBppm1RB was digested with *Asc*I/*Pac*I, and the digested DNA fragment containing the *Acppm1* downstream sequence was subcloned into pAgHBppm1LB to generate pAgHBppm1LR. Finally, pAgHBppm1LR was introduced into WT through ATMT. After selected on the TSA medium supplemented with 50 μg/mL hygromycin B or 10 μg/mL bleomycin, the hygromycin B resistant and bleomycin sensitive transformants were selected as Acppm1DM. Acppm1DM was identified by PCR with primers Acppm1Out-F/R and Acppm1Int-F/R, and further confirmed by Southern hybridization using the non-radioactive digoxigenin DNA labeling and Detection kit (Roche, Germany). For Southern hybridization, a 735 bp DNA fragment amplified from WT with primers Sppm1-F/R was used as probe and the fungal genomic DNA was digested with *Xho*I.

For complementation of Acppm1DM, a 3530 bp DNA fragment containing the entire *Acppm1* with its putative promoter and terminator regions was amplified with primers Acppm1C-F/R. Then the amplified DNA fragment was cloned into pEASY-Blunt to give pEBppm1C and verified by sequencing. The pEBppm1C was digested with *BamH*I and the fragment containing *Acppm1* was ligated into the corresponding site of pAgB. The resulting plasmid pAgB::Acppm1C was introduced into Acppm1DM through ATMT. The transformants were selected on the TSA medium with 10 μg/mL bleomycin and 400 μg/mL cefotaxime. The bleomycin resistant transformants were selected and identified as the complemented strains by PCR with primers RT-Acppm1-F/R. One complemented strain (Acppm1CM) was selected randomly and used for subsequent experiments.

To overexpress *Acppm1*, the entire *Acppm1* was amplified with primers Acppm1OE-F/R from the cDNA of *A. chrysogenum* and cloned into pEASY-Blunt to generate pEBppm1OE. After verified by sequencing, pEBppm1OE was digested with *Sma*I and the resulting DNA fragment containing *Acppm1* was ligated into the corresponding site of pAgH-Pgpd to give pAgHP::Acppm1. Finally, pAgHP::Acppm1 was introduced into WT through ATMT. The transformants were selected on the TSA medium with 50 μg/mL hygromycin B and 400 μg/mL cefotaxime. One hygromycin B resistant strain was selected as the *Acppm1* overexpressed strain (Acppm1OE) after confirmed by real-time RT-PCR with primers RT-Acppm1-F/R.

To overexpress *mecB*, the *mecB* gene was amplified with primers mecBOE-F/R from the cDNA of *A. chrysogenum* and cloned into pEASY-Blunt to give pEBmecBOE. After verified by sequencing, pEBmecBOE was digested with *Asc*I/*Pac*I. The digested DNA fragment containing *mecB* was cloned into the corresponding site of pAg1PT-G418. The resulting plasmid pAg1PT-G418::mecB was finally introduced into Acppm1DM through ATMT. The G418 resistant transformants were selected on the TSA medium with 250 μg/mL G418 and 400 μg/mL cefotaxime, one of them was randomly selected as the *mecB* overexpressed strain (Acppm1DM-mecBOE), and verified by real-time RT-PCR with primers RT-mecB-F/R.

To overexpress *AcsamS* in Acppm1DM-mecBOE, a fragment contained the entire bleomycin resistant gene (*ble*) together with promoter and terminator was amplified from the plasmid pJL43-RNAi with phosphorylated primers ble-F/R. After verified by sequencing, the fragment containing *ble* was subcloned into the plasmid pAg1PT-G418::AcsamS, generating pAg1PT-G418-AcsamS-ble. The resulting plasmid was finally introduced into Acppm1DM-mecBOE through ATMT. The bleomycin resistant transformants were selected on the TSA medium with 10 μg/mL bleomycin and 400 μg/mL cefotaxime as the *AcsamS* overexpressed strain (Acppm1DM-mecBOE-AcsamS), and verified by real-time RT-PCR with primers RT-Acsams-F/R. One of them was chosen randomly and used in subsequent experiments.

### Quantifying the intracellular concentration of SAM in *A. chrysogenum*

For measuring the intracellular concentration of SAM, the fungal strains of *A. chrysogenum* were incubated at 28 °C in MDFA medium. The fungal mycelia were collected after 3, 4 and 5 days fermentation, respectively. After dried with filter paper, the fungal mycelia were ground in liquid nitrogen using sterilized mortar and pestle. The trituration was dissolved in phosphate buffer saline (PBS) and the final protein concentration in the mixture was 30 μg/μL. The SAM concentration was quantified by the Enzyme-linked Immunosorbent assay (ELISA) kit for SAM detection (Cloud-clone corp., USA) according to the commercial manual.

### Detection of CPC production and fungal mycelium dry weight

For detection of CPC production, 3 × 10^7^ spores from WT, AcsamsOE, Acppm1DM, Acppm1CM, Acppm1OE and Acppm1DM-mecBOE were inoculated into 40 mL of seed medium (MDFA with methionine) in 250 mL flask, respectively. After incubated at 28 °C and 220 rpm for 2 days, 4 mL of seed culture was inoculated into 40 mL of MDFA medium supplemented with varying concentrations of methionine or SAM. The fermentation was carried out at 28 °C and 220 rpm for 7 days. Fungal mycelium dry weight was detected as described previously [20]. The CPC production was determined by bioassays against *B. subtilis* CGMCC 1.1630 with agar-diffusion method [20]. To exclude the accumulated penicillin N in the fermentation cultures, each LB test plate was added 50,000 units of penicillinase [23]. Standard of CPC-Zn (Sigma, USA) was used as control. The CPC production also measured with UPLC/MS by ACQUITY UPLC I-Class/Waters Xevo G2-XS QT system equipped with an electrospray ionization (ESI) source. The conditions used in this system were as follows: ACQUITY UPLC HSS T3 Column; 2.1 × 100 mm; 1–20% ACN in H_2_O over 6 min; 0.3 mL/min; 25 °C.

## Results and discussion

### Exogenous methionine stimulates CPC production through enhancing the intracellular *S*-adenosylmethionine of *A. chrysogenum*

**Fig. 2:**
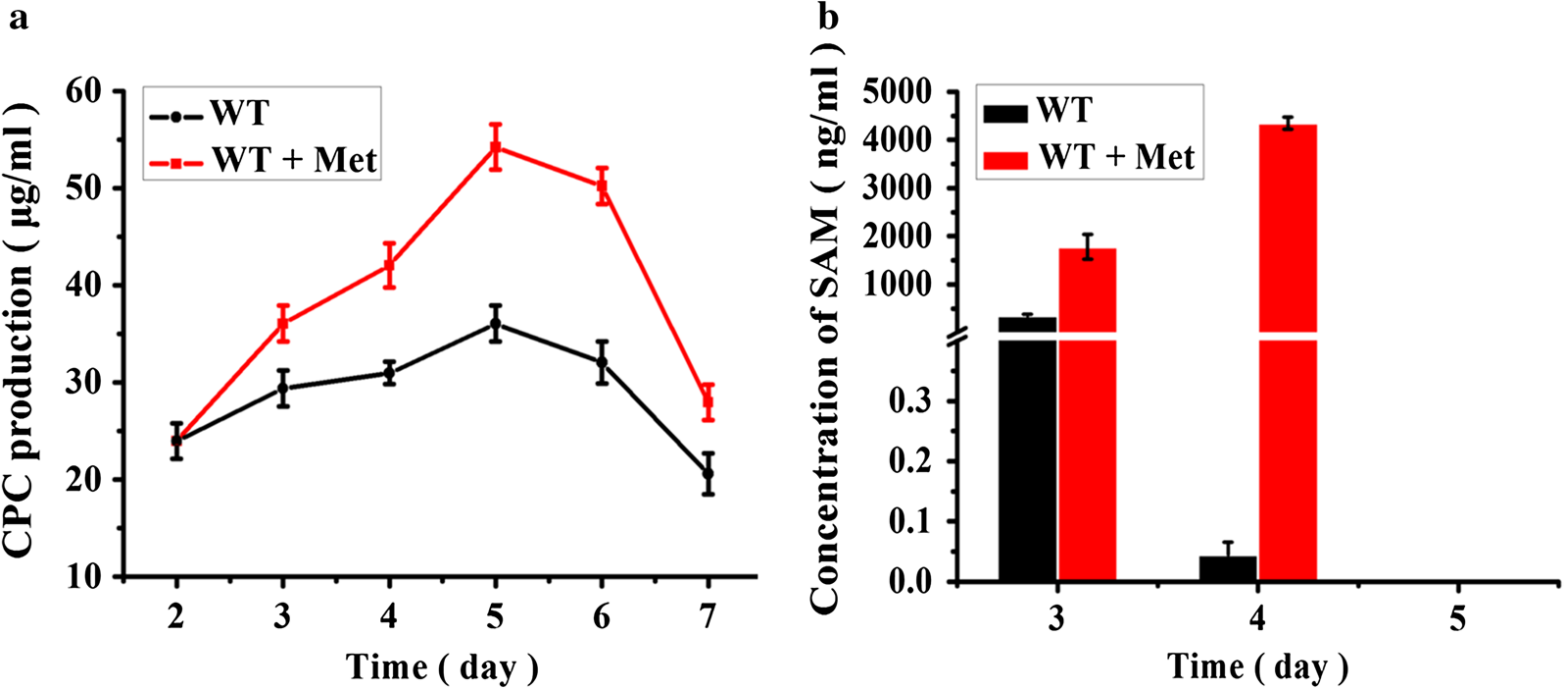
CPC production and intracellular concentration of SAM in *A. chrysogenum* during fermentation. **a** CPC production of *A. chrysogenum* with or without addition of 3.2 g/L methionine. The CPC production of WT grown in MDFA with or without addition of methionine was determined by bioassays against *B. subtilis* 1.1630 as described in “Methods”. WT, the *A. chrysogenum* wild-type strain; Met, methionine. **b** Intracellular concentrations of SAM in *A. chrysogenum* during fermentation with or without addition of 3.2 g/L methionine. The concentration of SAM was determined with the Enzyme-linked Immunosorbent Assay (ELISA) kit as described in “Methods”. The concentration of total protein was 30 μg/μL. No SAM could be detected in both cultures after 5 days fermentation with or without addition of methionine. Error bars represent standard deviations from three independent experiments

Methionine, especially the DL isomer, significantly stimulates CPC production of *A. chrysogenum* [24]. When the fermentation culture was supplemented with 3.2 g/L of dl-Methionine, the CPC production increased nearly two times (reached to 55 µg/mL) (Fig. 2a). To further confirm it, the CPC production was determined with UPLC/MS and the result showed that the quantitative signal intensity of CPC from the fermentation with 3.2 g/L methionine was stronger than that without methionine (Additional file 1: Fig. S1). Since methionine is the precursor of SAM which has been proved to stimulate antibiotic production in bacteria [25], the intracellular SAM concentration of *A. chrysogenum* was measured during fermentation in the MDFA medium with or without addition of methionine. The result showed that the intracellular concentration of SAM dropped remarkably after 4 days fermentation without addition of exogenous methionine, probably the endogenous methionine as the precursor has been exhausted at this time. However, the concentration of SAM remained and even increased by addition of exogenous methionine after 4 days fermentation (Fig. 2b). The SAM accumulation in *A. chrysogenum* with and without addition of exogenous methionine showed significant different after 4 days fermentation when CPC began to be produced. It is possible that the accumulation of intracellular SAM promoted by addition of exogenous methionine results in the increment of CPC production in *A. chrysogenum*. However, no SAM could be detected in both cultures after 5 days fermentation with or without addition of methionine. To eliminate the possibility of growth effect on CPC production, the fungal mycelium dry weight was also measured. The result showed that the growth of WT reached to the stationary phase at the second day of fermentation. However, the fungal growth even showed a slight decrease when addition of exogenous methionine (Additional file 1: Fig. S2). Therefore, the methionine stimulation on CPC production is due to the accumulation of intracellular SAM rather than growth of *A. chrysogenum*. Based on this consideration, we tried to construct a strain for improving CPC production independent of methionine stimulation through increasing the intracellular SAM concentration. For this purpose, the strategies through enhancing the SAM biosynthesis (“open source”) and blocking the SAM consumption (“throttling control”) were used.

### Cloning and characterization of *AcsamS* of *A. chrysogenum*

To increase the intracellular SAM concentration by “open source”, the SAM synthetase involved in methionine cycle of *A. chrysogenum* was considered as the key point. The SAM synthetase has been studied for a long time since it catalyzes the synthesis of SAM using methionine and ATP [14]. In *Streptomyces*, overexpression of the SAM synthetase coding gene (*metK*) enhanced novobiocin production and the intracellular SAM concentration [25]. By searching the *A. chrysogenum* genomic database using BLAST program, an open reading frame (ORF) coding the SAM synthetase was identified and designated *AcsamS* (GenBank Accession No. MG356328). Comparing the sequence of *AcsamS* cDNA and its genomic DNA, no intron was found. The theoretical molecular weight of the deduced protein AcSAMS is 43.45 kDa which contains 395 amino acids with a highly conserved SAM synthetase domain from 14 to 393 amino acid residues. Sequence alignment showed that AcSAMS shares highly homology with SAM synthetases from different species (Additional file 1: Fig. S3a). It shows 79% identity to PcSAMS (GenBank Accession No. KZN83839.1) from *Penicillium chrysogenum*, 74% identity to ScSAMS2 (GenBank Accession No. NP_010790.3) from *Saccharomyces cerevisiae*, 71% identity to HsSAMS1 (GenBank Accession No. NP_000420.1) from *Homo sapiens*, 62% identity to AtSAMS (GenBank Accession No. NP_188365.1) from *Arabidopsis thaliana*, 56% identity to SgmetK (GenBank Accession No. WP_037656538.1) from *Streptomyces griseofuscus*, 86% identity to FlSAMS (GenBank Accession No. KPA42556.1) from *Fusarium langsethiae*, 84% identity to NcSAMS (GenBank Accession No. XP_011392995.1) from *N. crassa.* Phylogenetic analysis showed AcSAMS is close to NcSAMS and FlSAMS (Additional file 1: Fig. S3b). The evolutionary conservation of AcSAMS implied it plays a significant role in methionine cycle of *A. chrysogenum*.

### Overexpression of *AcsamS* enhances CPC production and reduces its dependence on methionine stimulation

The CPC production of WT/pAg1PT-G418 (as the control strain) was similar to WT with or without exogenous methionine (Additional file 1: Fig. S4). The results indicated that pAg1PT-G418 can be used to construct overexpression strain. Therefore, the plasmid pAg1PT-G418::AcsamS was constructed and introduced into WT via ATMT to generate the *AcsamS* overexpressed strain (AcsamsOE). One *AcsamS* overexpressed strain was randomly selected and confirmed by real-time RT-PCR. The real-time RT-PCR showed that transcriptional level of *AcsamS* was increased 2.5-fold in AcsamsOE compared with that of WT (Additional file 1: Fig. S5).

**Fig. 3:**
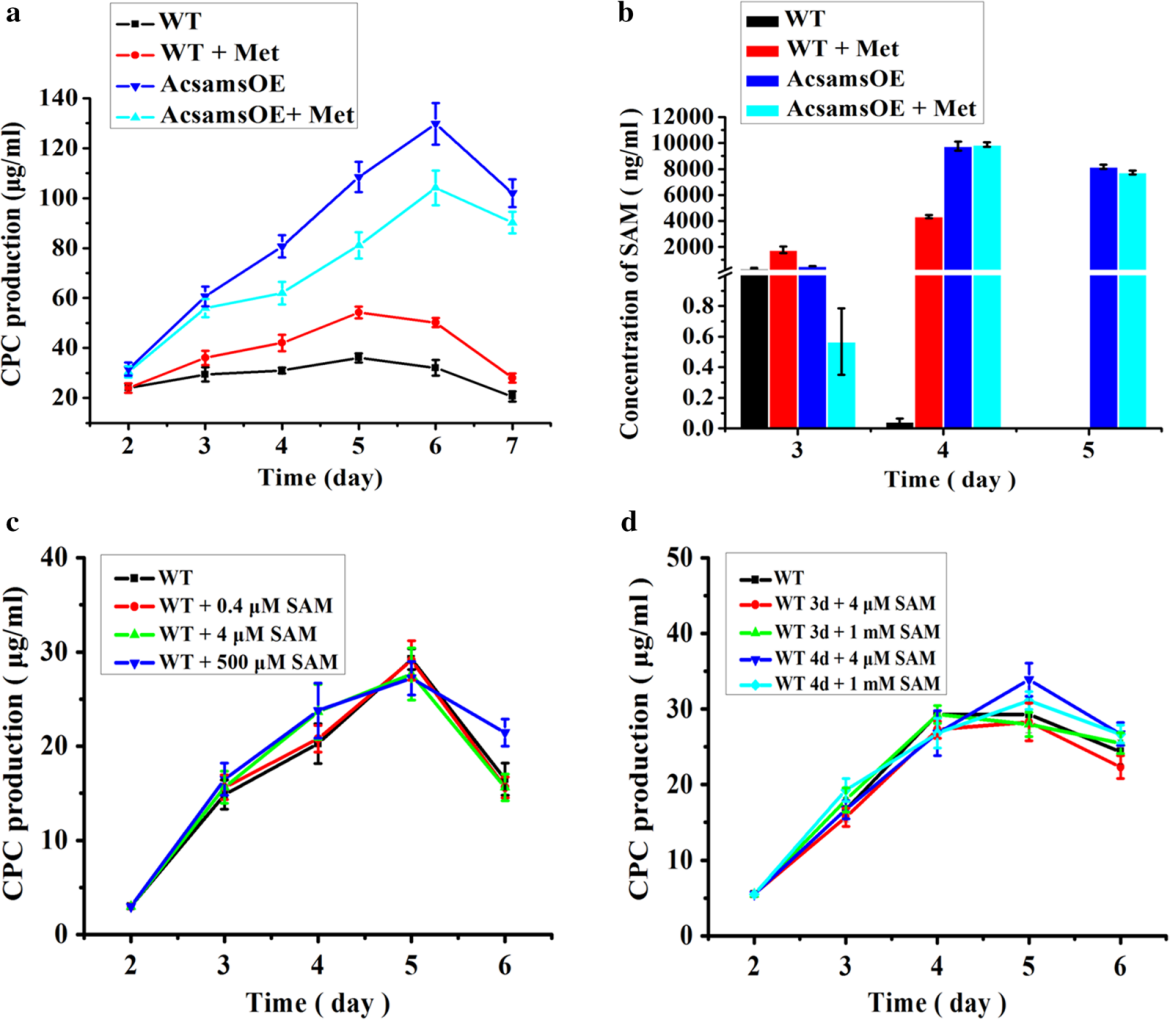
CPC production and intracellular concentration of SAM in WT and AcsamsOE. **a** CPC production in WT and AcsamsOE. CPC production of WT and AcsamsOE was determined by bioassays against *B. subtilis* 1.1630 as described in “Methods”. 3.2 g/L methionine was added in the MDFA medium when needed. **b** Intracellular concentrations of SAM in WT and AcsamsOE. Intracellular concentrations of SAM in WT and AcsamsOE were determined with the Enzyme-linked Immunosorbent Assay (ELISA) kit as described in “Methods”. The final concentration of methionine in the MDFA medium was 3.2 g/L. The concentration of total protein was 30 μg/μL. **c** CPC production of WT in the MDFA medium supplemented with 0, 0.4, 4.0 and 500 μM of SAM, respectively. **d** CPC production of WT in the MDFA medium supplemented with 4 μM and 1 mM of SAM after 3 or 4 days fermentation. The CPC production was determined by bioassays against *B. subtilis* 1.1630 as described in “Methods”. WT, the *A. chrysogenum* wild-type strain; AcsamsOE, the *AcsamS* overexpressed strain; Met, methionine; *SAM S*-adenosylmethionine. Error bars represent standard deviations from three independent experiments

As expected, AcsamsOE produced more CPC than WT. As shown in Fig. 3a, the CPC production (129.7 µg/mL) of AcsamsOE was approximately fourfold higher than that (36 µg/mL) of WT in the MDFA medium without addition of exogenous methionine. However, the CPC production of AcsamsOE was only 104 µg/mL when the fermentation culture was supplemented with exogenous methionine. It could be the repression of sulfate on CPC production caused by higher concentration of exogenous methionine [26]. The CPC production was also confirmed with UPLC/MS and the result showed that the quantitative signal intensity of CPC from AcsamsOE was stronger than that from WT (Additional file 1: Fig. S6). Except the second day of fermentation, the mycelium dry weight of WT was higher than that of AcsamsOE in the MDFA medium either with or without addition of 3.2 g/L methionine (Additional file 1: Fig. S7). Therefore the increase of CPC production in AcsamsOE was mainly due to the overexpression of *AcsamS*.

To further address the reason, the intracellular SAM concentration of AcsamsOE was measured after fermentation in the MDFA medium with or without addition of exogenous methionine. As expected, the intracellular SAM concentration of AcsamsOE was enhanced significantly in both situations, but addition of exogenous methionine in AcsamsOE did not further increase the intracellular SAM concentration (Fig. 3b), indicating that the endogenous methionine was either enough for the synthesis of SAM or the extra methionine could suppress the synthesis of SAM in AcsamsOE, this could also be the reason that the concentration of SAM was very low in AcsamsOE at 3rd day of fermentation when the exogenous methionine was added. On the other hand, it was also validated the importance of the endogenous methionine for CPC production as reported previously [27]. Not like WT in which the SAM concentration declined dramatically after 4 days fermentation, AcsamsOE almost remained the same level of SAM concentration even after 5 days fermentation (Fig. 3b). The increment of CPC production in AcsamsOE could be due to the accumulation of intracellular SAM, especially at the late stage of fermentation. These results indicated the dependence of methionine stimulation during CPC production could be significantly reduced by enhancing the intracellular SAM in the metabolic engineered strain.

### Exogenous SAM supplementation does not increase CPC production

Since accumulation of the intracellular SAM concentration through “open source” could significantly increase CPC production, we tried to add exogenous SAM into the fermentation culture of *A. chrysogenum* to improve CPC production just like the fact that addition of exogenous SAM led to an increase of antibiotic production in *S. coelicolor* M145 [18]. However, addition of exogenous SAM in the fermentation culture of *A. chrysogenum* with varying concentrations (0.4, 4 and 500 µM, respectively) didn’t increase the CPC production (Fig. 3c). We also checked the effect of exogenous SAM added after 3 or 4 days fermentation, the CPC production didn’t increase even with addition of 1 mM exogenous SAM (Fig. 3d). In AcsamsOE, the similar result was found that addition of the exogenous SAM didn’t further increase the CPC production (Additional file 1: Fig. S8). It was reported that SAM could be rapidly consumed in animal cells [28]. Combined with the fact that the intracellular SAM of AcsamsOE was detected even after 5 days fermentation since the transcription of *AcsamS* continuously remained at high level, we suspected that the exogenous SAM could be quickly consumed since the exogenous SAM stimulates expression of SAM dependent methyltransferase mainly responsible for SAM consumption.

### Transcriptional analysis of the putative SAM dependent methyltransferase encoding genes in *A. chrysogenum*

**Fig. 4:**
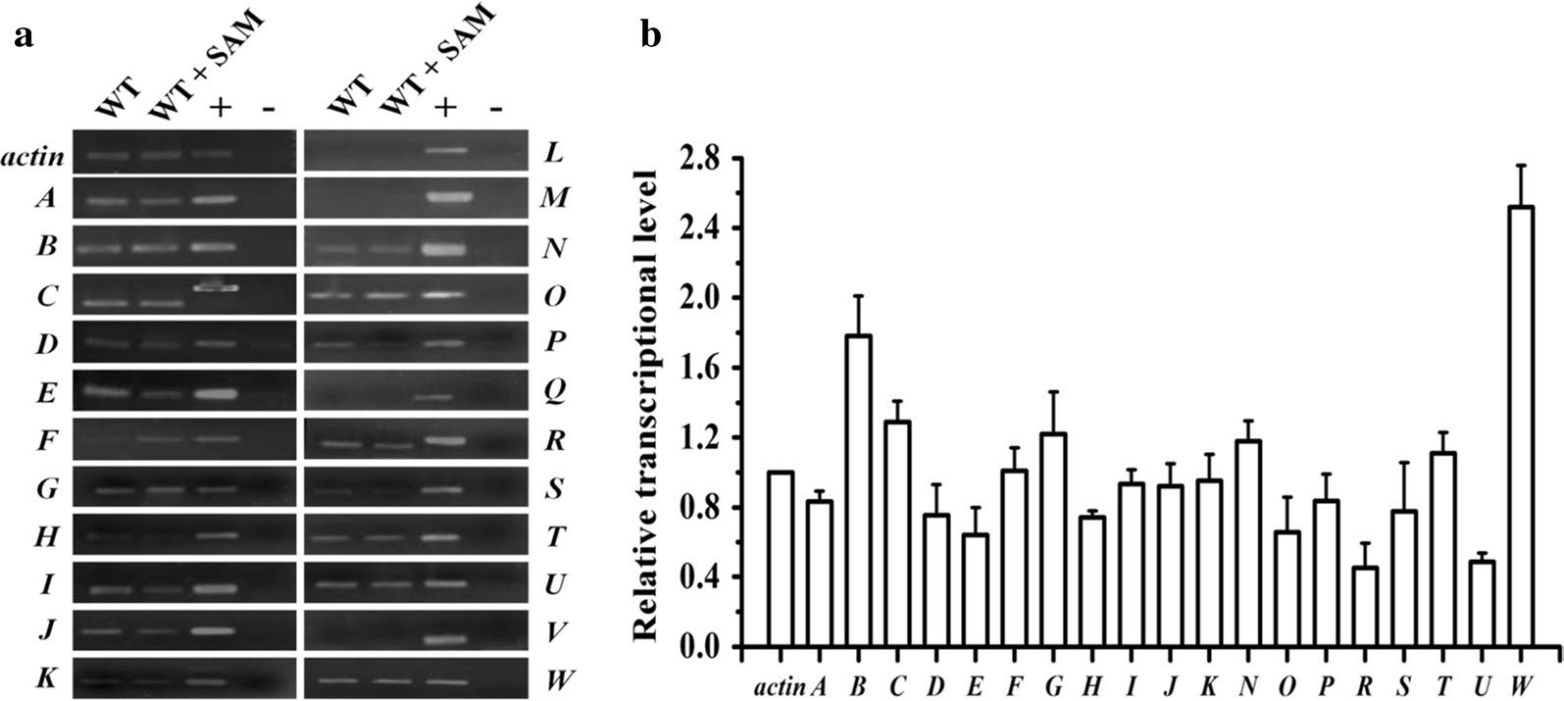
Transcription of 23 putative SAM dependent methyltransferase encoding genes (*AcMTA*-*W*) in *A. chrysogenum*. **a** Transcriptional analysis of *AcMTA*-*W* by semi-quantitation RT-PCR. Total RNA extraction and cDNA synthesis were performed as described in “Methods”. WT, the amplified products using cDNA isolated from WT which was grown in the MDFA medium at 28 °C for 5 days; WT + SAM, the amplified products using cDNA isolated from WT which was grown at 28 °C for 5 days and 500 μM of SAM was added in the MDFA medium at 4th day; +, the genomic DNA from WT as the positive control; −, the negative control. Transcription of the *actin* gene was used as control. **b** Transcriptional analysis of *AcMTA*-*W* by real-time RT-PCR. WT was grown at 28 °C for 5 days in the MDFA medium with or without addition of 500 μM of SAM after 4 days fermentation. Total RNA extraction and cDNA synthesis were performed as described in “Methods”. The relative transcriptional level of *AcMTA*-*W* was detected by real-time RT-PCR. The relative abundance of mRNAs was standardized against the transcriptional level of *actin* gene. *A*-*W*, the transcriptions of putative SAM dependent methyltransferase encoding genes *AcMTA*-*W*; *actin*, the transcription of *actin* gene. Error bars represent standard deviations from three independent experiments

SAM, as the principal biological methyl donor, is utilized mainly for the methylation reaction through methyltransferases [15]. It has been reported that a glycine N methyltransferase (GNMT) acts as a “cellular buffer” which maintains the intracellular concentration of SAM by consuming the redundant SAM in mice [29, 30]. We suspected that the intracellular redundant SAM of *A. chrysogenum* could be consumed by the similar pathway in which some SAM dependent methyltransferases play a key role. In search of the *A. chrysogenum* genomic database, 23 putative SAM dependent methyltransferase encoding genes were identified using BLAST program and designated *AcMTA*-*W*, respectively. To determine which methyltransferase plays the key role in SAM consumption, the transcriptions of *AcMTA*-*W* in WT grown in the MDFA medium with or without addition of exogenous SAM were measured through semi-quantitation RT-PCR and real-time RT-PCR. As shown in Fig. 4a, most of the methyltransferase encoding genes transcribed in WT except *AcMTL*, *AcMTM*, *AcMTQ* and *AcMTV*. The real-time RT-PCR result demonstrated that the transcriptional level of *AcMTB*, *AcMTC*, *AcMTG*, *AcMTN*, *AcMTT* and *AcMTW* was clearly increased when the fermentation culture was added with 500 µM of SAM. Of them, the increment of *AcMTW* transcriptional level was the maximum (Fig. 4b). Thus, we assumed that the protein encoded by *AcMTW* is the major consumer of SAM and responsible for maintaining the normal intracellular SAM concentration in *A. chrysogenum*.

We renamed *AcMTW* as *Acppm1* (GenBank Accession No. MG356326). Comparing the sequence of *Acppm1* cDNA and genomic DNA, a 78 bp intron was identified. Theoretical molecular weight of the deduced AcPPM1 is 42.13 kDa. AcPPM1 contains 383 amino acid residues with a SAM dependent methyltransferase conserved domain. Sequence alignment showed that AcPPM1 shares highly homology with leucine carboxyl methyltransferase superfamily proteins. It shows 63% identity to leucine carboxyl methyltransferase 1 (GenBank Accession No. XP_011323584.1) from *Fusarium graminearum*, 61% identity to leucine carboxyl methyltransferase 1 (GenBank Accession No. KPM38892.1) from *Neonectria ditissima*, 56% identity to leucine carboxyl methyltransferase 1 (GenBank Accession No. PHH53006.1) from *Ceratocystis fimbriata*, 58% identity to leucine carboxyl methyltransferase 1 (GenBank Accession No. XP_016641023.1) from *Scedosporium apiospermum*, 57% identity to leucine carboxyl methyltransferase 1 (GenBank Accession No. KKP05041.1) from *Trichoderma harzianum* (Additional file 1: Fig. S9). However, the functions of all these proteins are unknown.

### Disruption of *Acppm1* enhances CPC production and reduces its dependence on methionine stimulation

Since the hepatic SAM concentration was increased about 40-fold in the leucine carboxyl methyltransferase gene (*GNMT*) knock-out mice [29, 30], we assumed that disruption of *Acppm1* could accumulate the intracellular SAM of *A. chrysogenum*. The strategy for accumulating SAM by blocking its consumption is called “throttling control”.

For increasing the intracellular SAM concentration, the plasmid pAgHBppm1LR was introduced into WT via ATMT to generate the *Acppm1* disruption mutant (Acppm1DM) through homologous recombination (Additional file 1: Fig. S10a). One *Acppm1* disruption mutant was isolated randomly and confirmed by PCR. The 2092 and 2992 bp DNA fragments were amplified with the primers outside *Acppm1* from WT to Acppm1DM, respectively. When using the primers inside *Acppm1*, a 673 bp fragment was amplified only from WT but not from Acppm1DM (Additional file 1: Fig. S10b). To exclude ectopic integrations of the resistant cassette into the fungal genome, Southern hybridization was further performed. As expected, a 5785 bp fragment was detected in Acppm1DM while a 3995 bp fragment was detected in WT (Additional file 1: Fig. S10c). These results indicated that *Acppm1* was replaced by the hygromycin phosphotransferase gene (*hph*) in Acppm1DM. RT-PCR analysis revealed the transcription of *Acppm1* was completely abolished in Acppm1DM, while its transcription was restored in the complemented strain Acppm1CM (Additional file 1: Fig. S10d). The *Acppm1* overexpressed strain (Acppm1OE) was also constructed and confirmed by real-time RT-PCR. The transcriptional level of *Acppm1* was increased nearly 25-fold in Acppm1OE (Additional file 1: Fig. S10e).

**Fig. 5:**
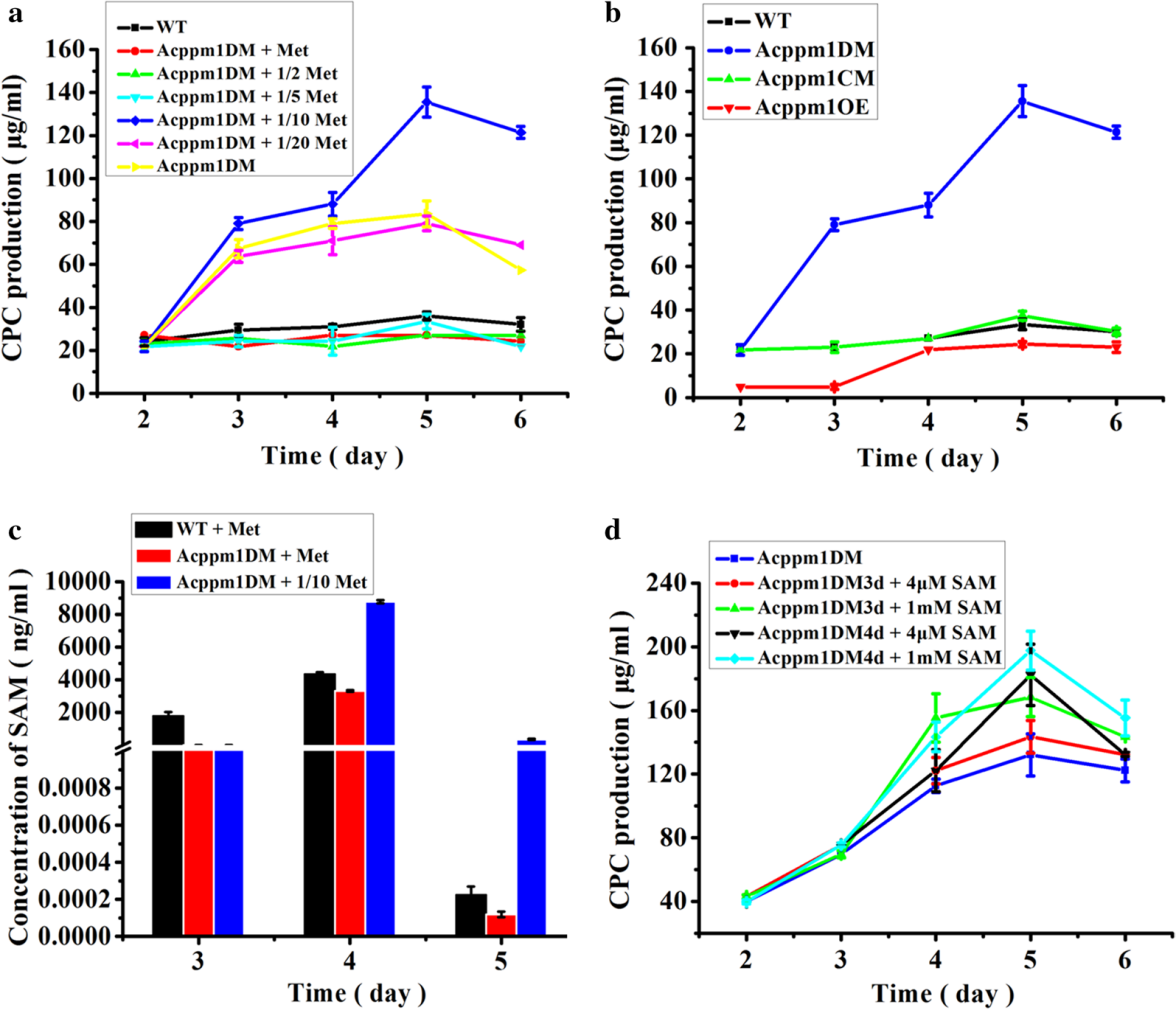
CPC production and intracellular SAM concentration in Acppm1DM. **a** CPC production of Acppm1DM in the MDFA medium supplemented with varying concentrations of methionine. One half (1.6 g/L), one-fifth (0.64 g/L), one-tenth (0.32 g/L), one-twentieth (0.16 g/L) and the normal concentration of methionine (3.2 g/L) were used in the MDFA medium for fermentation. **b** CPC production of WT, Acppm1DM, Acppm1CM and Acppm1OE in the MDFA medium supplemented with 0.32 g/L methionine. **c** Intracellular SAM concentration of WT and Acppm1DM. The concentrations of methionine supplemented in the MDFA medium were 3.2 g/L and 0.32 g/L, respectively. **d** CPC production of Acppm1DM with addition of exogenous SAM during fermentation. 4 μM and 1 mM of SAM were added after 3 or 4 days fermentation. Acppm1DM3d, SAM was added after 3 days fermentation; Acppm1DM4d, SAM was added after 4 days fermentation. WT, the wild-type strain; Acppm1DM, the *Acppm1* disruption mutant; Acppm1CM, the complemented strain of Acppm1DM with a copy of *Acppm1*; Acppm1OE, the *Acppm1* overexpressed strain. Met, methionine. CPC production was determined by bioassays against *B. subtilis* 1.1630 as described in the “Methods”. Error bars represent standard deviations from three independent experiments

To detect the stimulation of methionine on CPC production in Acppm1DM, the modified MDFA medium supplemented with varying concentrations of methionine was used during fermentation. As shown in Fig. 5a, Acppm1DM showed the highest CPC production (135.5 µg/mL) when the concentration of supplied methionine in MDFA medium was reduced to 0.32 g/L (one-tenth of the normal level). However, the CPC yield of Acppm1DM was almost the same as that of WT in the MDFA medium with normal concentration of methionine (3.2 g/L). When the methionine concentration in MDFA medium was less than 0.32 g/L, the CPC yield of Acppm1DM was significantly decreased. Therefore, disruption of *Acppm1* resulted in the increment of CPC production and significantly reduced the dependence of methionine stimulation on CPC production. The MDFA medium supplemented with 0.32 g/L methionine could be the optimum condition for CPC production in Acppm1DM. In agreement with above results, the CPC production in the complemented strain (Acppm1CM) restored to the wild-type level in the MDFA medium supplemented with 0.32 g/L methionine and overexpression of *Acppm1* significantly inhibited CPC production (Fig. 5b). The UPLC/MS result also demonstrated that the quantitative signal intensity of CPC from Acppm1DM in the MDFA medium with addition of 0.32 g/L methionine was much stronger than that from WT in the MDFA medium without methionine (Additional file 1: Fig. S11). Meanwhile, the mycelium dry weight of Acppm1DM was almost the same as that of WT and Acppm1CM (Additional file 1: Fig. S12), indicating that the increment of CPC production in Acppm1DM was due to the disruption of *Acppm1*.

The concentration of intracellular SAM was also quantified and discovered that the SAM concentration in Acppm1DM was twofold higher than that in WT when 0.32 g/L methionine was added in the MDFA medium. With 0.32 g/L methionine in the fermentation medium, the SAM concentration in Acppm1DM reached to a maximum level after 4 days fermentation and retained high level even after 5 days fermentation. While, the SAM concentration of WT was decreased to a very low level after 5 days fermentation, it may be caused by the deficiency of methionine (Fig. 5c). When 3.2 g/L methionine was added in the MDFA medium, the intracellular SAM decreased sharply in Acppm1DM (Fig. 5c). To assess the possible reason for this phenomenon, we detected the transcriptional level of the *AcsamS* in WT and Acppm1DM. The result showed that addition of exogenous methionine led to a reduction of *AcsamS* transcription (Additional file 1: Fig. S13a, b), the same as reported before in human hepatocarcinoma cells [31]. Furthermore, the transcriptional level of *AcsamS* in Acppm1DM was obviously lower than that in WT with 3.2 g/L methionine in the fermentation medium (Additional file 1: Fig. S13c). Thus it was possible that inhibition of SAM biosynthesis by adding 3.2 g/L methionine caused a decrease of SAM concentration in Acppm1DM. These results indicated that the main reason for CPC increment in Acppm1DM was the intracellular SAM accumulation.

We further measured the effect of exogenous SAM on CPC production in Acppm1DM. As expected, the CPC production was improved further by adding SAM (4 µM and 1 mM, respectively) after 3 or 4 days fermentation. High concentration of SAM showed more effective on the stimulation of CPC production. When adding SAM after 4 days fermentation, the CPC production was higher than that when adding SAM after 3 days fermentation (Fig. 5d). These results suggested that AcPpm1 was the major consumer of intracellular SAM and we could enhance the CPC production with only 0.32 g/L methionine through “throttling control”.

To find the possible pathway from SAM to CPC in *A. chrysogenum*, we detected the transcriptional level of the related genes in methionine cycle during fermentation with or without addition of exogenous methionine. By searching the *A. chrysogenum* genomic database using BLAST program, four open reading frames (ORFs) coding the homocysteine synthase, cystathionine-β-synthase, cystathionine-γ-lyase and methionine synthase (Fig. 1) were identified and designated as *AccysD*, *AcmecA*, *mecB* and *AcmetH*, respectively. The real-time RT-PCR results showed that the transcription of *AcmetH* was decreased when exogenous methionine was added in MDFA medium (Additional file 1: Fig. S14a). However, it restored after 5 days fermentation probably due to running out of methionine in MDFA medium. This result indicated that exogenous methionine inhibits the synthesis of endogenous methionine. If exogenous methionine is exhausted, the endogenous methionine was widely synthesized. Whereas the transcriptional level of *AccysD*, *AcmecA*, and *mecB* were increased during fermentation with addition of methionine (Additional file 1: Fig. S14b–d), indicating that the exogenous methionine can promote the metabolic flux towards l-cysteine synthesis. Consequently, one of the main reasons for enhancing CPC production through SAM accumulation may be the increase of the CPC precursor l-cysteine.

### Overexpressing *mecB* in Acppm1DM leads to improvement of CPC production independent of methionine stimulation

Since enhancing the intracellular SAM concentration of *A. chrysogenum* increased CPC production, we tried to improve the intracellular SAM concentration further through overexpressing *AcsamS* in Acppm1DM. The *AcsamS* overexpressed strain (Acppm1DM-AcsamsOE) was constructed through introducing pAg1PT-G418::AcsamS into Acppm1DM. However, the CPC production of Acppm1DM-AcsamsOE was not increased and even lower than that of Acppm1DM (Additional file 1: Fig. S15a). When increasing the concentration of methionine in MDFA medium, the CPC production of Acppm1DM-AcsamsOE even decreased (Additional file 1: Fig. S15b).

**Fig. 6:**
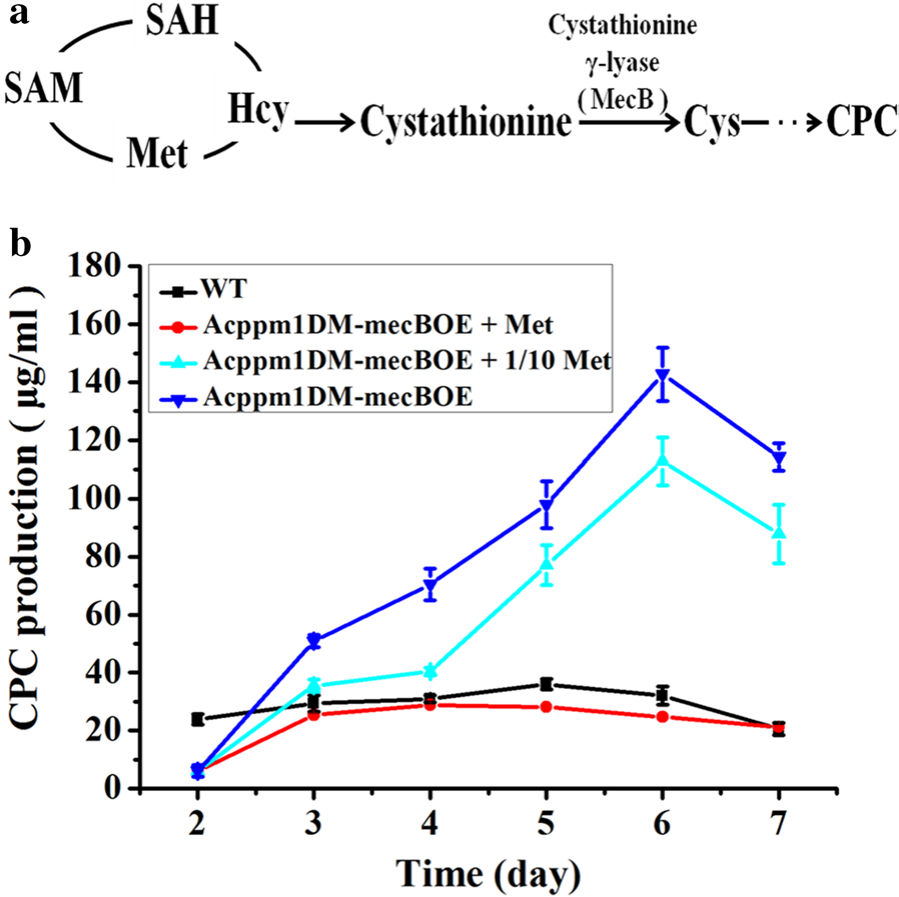
CPC production of Acppm1DM-mecBOE in the MDFA medium supplemented with varying concentrations of methionine. **a** The streamlined diagram of methionine cycle and cephalosporin biosynthesis. *Hcy* homocysteine, *Cys* cysteine, *SAH S*-adenosylhomocysteine, *SAM S*-adenosylmethionine, *Met* methionine, *CPC* cephalosporin C. The conversion of cystathionine to cysteine is catalyzed by cystathionine-γ-lyase (MecB). **b** The CPC production of Acppm1DM-mecBOE in the MDFA medium supplemented with 0, 0.32 and 3.2 g/L of methionine, respectively. CPC production was determined by bioassays against *B. subtilis* 1.1630 as described in “Methods”. Acppm1DM-mecBOE, overexpressing *mecB* in Acppm1DM. Error bars represent standard deviations from three independent experiments

It has been reported that cystathionine-γ-lyase (catalyzed the cystathionine to l-cysteine as shown in Fig. 6a) was required for high-level production of CPC and overexpression of the cystathionine-γ-lyase gene *mecB* enhanced the CPC production 10–40% in the *A. chrysogenum* strain C10 [32, 33]. Unlike the wild-type *A. chrysogenum* (Brotzu’s strain) and C10 in which the transcriptional level of *mecB* was not significantly affected by addition of exogenous methionine [34], our result showed that addition of exogenous methionine significantly stimulated the *mecB* transcription in both WT (Additional file 1: Fig. S14d) and Acppm1DM (Additional file 1: Fig. S16). Therefore, it is possible that the decrease of CPC production in Acppm1DM-AcsamsOE was due to the reduction of the *mecB* expression since no exogenous methionine was added in the fermentation medium.

To reduce the methionine dependence of CPC production, the plasmid pAg1PT-G418::mecB was constructed and transformed into Acppm1DM via ATMT. The resulting *mecB* overexpressed strain (Acppm1DM-mecBOE) was verified by real-time RT-PCR (Additional file 1: Fig. S17). One verified transformant was randomly selected for subsequent experiments. For fermentation, Acppm1DM-mecBOE was cultured in the MDFA medium supplemented with varying concentrations of methionine (0, 0.32 and 3.2 g/L respectively). After 6 days fermentation in the MDFA medium without addition of methionine, the CPC production of Acppm1DM-mecBOE reached to the maximum value (142.7 µg/mL), which was higher than that in the MDFA medium supplemented with 0.32 or 3.2 g/L of methionine (Fig. 6b). The result was also confirmed by UPLC/MS, the result demonstrated that the quantitative signal intensity of CPC from Acppm1DM-mecBOE was much stronger than that from WT in the MDFA medium without methionine (Additional file 1: Fig. S18). The growth of Acppm1DM-mecBOE was slower than that of WT at the exponential phase. At the third day, fungal growth reached to the stationary phase. During the stationary phase, Acppm1DM-mecBOE had a mycelium dry weight comparable to WT (Additional file 1: Fig. S19). The slower growth rate of Acppm1DM-mecBOE was consistent with the phenomenon of the strain T58 which showed a five to six fold higher cystathionine-γ-lyase activity than its parental strain [33]. These results clearly demonstrated that the CPC production improvement in Acppm1DM-mecBOE was independent of methionine stimulation.

Furthermore, the plasmid pAg1PT-G418-AcsamS-ble was constructed and transformed into Acppm1DM-mecBOE to obtain the *AcsamS* overexpressed strain Acppm1DM-mecBOE-AcsamsOE. After verified by real-time RT-PCR (Additional file 1: Fig. S20), one transformant was randomly selected and used for fermentation. For fermentation, the Acppm1DM-mecBOE-AcsamsOE was cultured in the MDFA medium with addition of varying concentrations of methionine (0, 0.32 and 3.2 g/L, respectively). During fermentation in the MDFA medium without methionine, the CPC production of Acppm1DM-mecBOE-AcsamsOE was almost the same as that of Acppm1DM-mecBOE (Additional file 1: Fig. S21), indicating that the expression of *mecB* is the rate-limiting step of the CPC production in Acppm1DM and Acppm1DM-AcsamsOE. Like in Acppm1DM-mecBOE, the CPC production in Acppm1DM-mecBOE-AcsamsOE was decreased in the MDFA medium with addition of methionine (Additional file 1: Fig. S21). Since the regulation of CPC biosynthesis is complicated and the intracellular SAM accumulation could inhibit expression of *AcsamS* or affect the activity of AcSAMS, Acppm1DM-AcsamsOE and Acppm1DM-mecBOE-AcsamsOE did not increased the CPC production further compared with Acppm1DM and Acppm1DM-mecBOE.

## Conclusions

Our results revealed that methionine stimulated CPC production through enhancing the intracellular SAM of *A. chrysogenum* and the CPC production could be improved by accumulating endogenous SAM during fermentation. Two strategies, including enhancement of SAM synthesis and blockage of SAM consumption, were used to overcome the dependence of methionine stimulation during CPC production. Finally, an optimum recombinant strain Acppm1DM-mecBOE was constructed through engineering the methionine cycle. In this strain, CPC production was increased 5.5-fold and its improvement was totally independent of methionine stimulation. This study provides a novel insight for improving CPC production independent of methionine stimulation.

## Additional file

**Additional file 1: Table S1.** Strains and plasmids used in this study. **Table S2.** Primers used in this study. **Fig. S1.** Cephalosporin C production of WT detected by UPLC/MS in the MDFA medium with or without addition of 3.2 g/L methionine. **Fig. S2.** Mycelium dry weight of *A. chrysogenum* in the MDFA medium with or without addition of 3.2 g/L methionine. **Fig. S3.** Sequence alignment and phylogenetic analysis of the SAM synthetase family proteins. **Fig. S4.** Cephalosporin C production of WT and WT/pAg1PT-G418 in the MDFA medium with or without addition of 3.2 g/L methionine. **Fig. S5.** Construction and validation of the *AcsamS* overexpressed strain (AcsamsOE). **Fig. S6.** Cephalosporin C production of WT and AcsamsOE was detected by UPLC/MS in MDFA medium. **Fig. S7.** Mycelium dry weight of AcsamsOE in the MDFA medium with or without addition of 3.2 g/L methionine. **Fig. S8.** Cephalosporin C production of AcsamsOE in the MDFA medium supplemented with different concentration of SAM. **Fig. S9.** Sequence alignment of the leucine carboxyl methyltransferase superfamily proteins. **Fig. S10.** Construction and validation of the *Acppm1* disruption mutant (Acppm1DM). **Fig. S11.** Cephalosporin C production of WT and Acppm1DM was detected by UPLC/MS. **Fig. S12.** Mycelium dry weight of Acppm1DM, Acppm1CM, Acppm1OE in the MDFA medium with or without addition of 0.32 g/L methionine. **Fig. S13.** The relative transcriptional level of *AcsamS* in WT and Acppm1DM. **Fig. S14.** The relative transcriptional level of *AcmetH*, *AccysD*, *AcmecA* and *mecB* of WT in the MDFA medium with or without addition of 3.2 g/L methionine. **Fig. S15.** Cephalosporin C production of Acppm1DM and Acppm1DM-AcsamsOE. **Fig. S16.** The relative transcriptional level of *mecB* in Acppm1DM. **Fig. S17.** Construction and validation of the *mecB* overexpressed strain (Acppm1DM-mecBOE). **Fig. S18.** Cephalosporin C production of WT and Acppm1DM-mecBOE was detected by UPLC/MS in MDFA medium. **Fig. S19.** Mycelium dry weight of WT and Acppm1DM-mecBOE in the MDFA medium supplemented with 0, 0.32 g/L and 3.2 g/L of methionine respectively. **Fig. S20.** Construction and validation of Acppm1DM-mecBOE-AcsamsOE. **Fig. S21.** Cephalosporin C production of Acppm1DM-mecBOE-AcsamsOE.

## Abbreviations

SAM: *S*-adenosylmethionine
CPC: cephalosporin C
SAH: *S*-adenosylhomocysteine
MTHF: methyltetrahydrofolate
THF: tetrahydrofolate
ATMT: *Agrobacterium tumefaciens*-mediated transformation
PBS: phosphate buffer saline
*hph*: the hygromycin phosphotransferase gene
Hcy: homocysteine
Cys: cysteine
Met: methionine
ACV: the tripeptide δ-(l-α-aminoadipyl)-L-cysteinyl-d-valine
