# Supplementary material for: Metabolic engineering of Acremonium chrysogenum for improving cephalosporin C production independent of methionine stimulation
Source: Microb Cell Fact. 2018 Jun 7;17:87. doi: 10.1186/s12934-018-0936-5 (PMC5992653; doi:10.1186/s12934-018-0936-5)
Supplement: Supplementary file 1 — Additional file 1: Table S1. Strains and plasmids used in this study. Table S2. Primers used in this study. Fig. S1. Cephalosporin C production of WT detected by UPLC/MS in the MDFA medium with or without addition of 3.2 g/L methionine. Fig. S2. Mycelium dry weight of A. chrysogenum in the MDFA medium with or without addition of 3.2 g/L methionine. Fig. S3. Sequence alignment and phylogenetic analysis of the SAM synthetase family proteins. Fig. S4. Cephalosporin C production of WT and WT/pAg1PT-G418 in the MDFA medium with or without addition of 3.2 g/L methionine. Fig. S5. Construction and validation of the AcsamS overexpressed strain (AcsamsOE). Fig. S6. Cephalosporin C production of WT and AcsamsOE was detected by UPLC/MS in MDFA medium. Fig. S7. Mycelium dry weight of AcsamsOE in the MDFA medium with or without addition of 3.2 g/L methionine. Fig. S8. Cephalosporin C production of AcsamsOE in the MDFA medium supplemented with different concentration of SAM. Fig. S9. Sequence alignment of the leucine carboxyl methyltransferase superfamily proteins. Fig. S10. Construction and validation of the Acppm1 disruption mutant (Acppm1DM). Fig. S11. Cephalosporin C production of WT and Acppm1DM was detected by UPLC/MS. Fig. S12. Mycelium dry weight of Acppm1DM, Acppm1CM, Acppm1OE in the MDFA medium with or without addition of 0.32 g/L methionine. Fig. S13. The relative transcriptional level of AcsamS in WT and Acppm1DM. Fig. S14. The relative transcriptional level of AcmetH, AccysD, AcmecA and mecB of WT in the MDFA medium with or without addition of 3.2 g/L methionine. Fig. S15. Cephalosporin C production of Acppm1DM and Acppm1DM-AcsamsOE. Fig. S16. The relative transcriptional level of mecB in Acppm1DM. Fig. S17. Construction and validation of the mecB overexpressed strain (Acppm1DM-mecBOE). Fig. S18. Cephalosporin C production of WT and Acppm1DM-mecBOE was detected by UPLC/MS in MDFA medium. Fig. S19. Mycelium dry weight of WT and Acppm1DM-mecBOE in the MDFA medium suppl [file 12934_2018_936_MOESM1_ESM.doc]

**Additional file 1**

**Metabolic engineering of *Acremonium chrysogenum* for improving cephalosporin C production independent of methionine stimulation**

Jiajia Liu^1,2^, Wenyan Gao^1^, Yuanyuan Pan^1^, Gang Liu^1,2^*

^1^State Key Laboratory of Mycology, Institute of Microbiology, Chinese Academy of Sciences, Beijing 100101, China

^2^University of Chinese Academy of Sciences, Beijing 100049, China

^*^ Correspondence

Email addresses of all authors:

Jiajia Liu: [jiajialiu0802@163.com](mailto:jiajialiu0802@163.com); Wenyan Gao: [gaowy@im.ac.cn](mailto:gaowy@im.ac.cn); Yuanyuan Pan: panyy@im.ac.cn; Gang Liu: [liug@im.ac.cn](mailto:liug@im.ac.cn)

**Content**

**Table S1** Strains and plasmids used in this study.

**Table S2** Primers used in this study.

**Fig. S1** Cephalosporin C production of WT detected by UPLC/MS in the MDFA medium with or without addition of 3.2 g/L methionine.

**Fig. S2** Mycelium dry weight of *A. chrysogenum* in the MDFA medium with or without addition of 3.2 g/L methionine.

**Fig. S3** Sequence alignment and phylogenetic analysis of the SAM synthetase family proteins.

**Fig. S4** Cephalosporin C production of WT and WT/pAg1PT-G418 in the MDFA medium with or without addition of 3.2g/L methionine.

**Fig. S5** Construction and validation of the *AcsamS* overexpressed strain (AcsamsOE).

**Fig. S6** Cephalosporin C production of WT and AcsamsOE was detected by UPLC/MS in MDFA medium.

**Fig.** **S7** Mycelium dry weight of AcsamsOE in the MDFA medium with or without addition of 3.2 g/L methionine.

**Fig. S8** Cephalosporin C production of AcsamsOE in the MDFA medium supplemented with different concentration of SAM.

**Fig. S9** Sequence alignment of the leucine carboxyl methyltransferase superfamily proteins.

**Fig. S10** Construction and validation of the *Acppm1* disruption mutant (Acppm1DM).

**Fig. S11** Cephalosporin C production of WT and Acppm1DM was detected by UPLC/MS.

**Fig. S12** Mycelium dry weight of Acppm1DM, Acppm1CM, Acppm1OE in the MDFA medium with or without addition of 0.32 g/L methionine.

**Fig. S13** The relative transcriptional level of *AcsamS* in WT and Acppm1DM.

**Fig. S14** The relative transcriptional level of *AcmetH*, *AccysD*, *AcmecA* and *mecB* of WT in the MDFA medium with or without addition of 3.2 g/L methionine.

**Fig. S15** Cephalosporin C production of Acppm1DM and Acppm1DM-AcsamsOE.

**Fig. S16** The relative transcriptional level of *mecB* in Acppm1DM.

**Fig. S17** Construction and validation of the *mecB* overexpressed strain (Acppm1DM-mecBOE).

**Fig. S18** Cephalosporin C production of WT and Acppm1DM-mecBOE was detected by UPLC/MS in MDFA medium.

**Fig. S19** Mycelium dry weight of WT and Acppm1DM-mecBOE in the MDFA medium supplemented with 0, 0.32 g/L and 3.2 g/L of methionine respectively.

**Fig. S20** Construction and validation of Acppm1DM-mecBOE-AcsamsOE.

**Fig. S21** Cephalosporin C production of Acppm1DM-mecBOE-AcsamsOE.

**Table S1** Strains and plasmids used in this study.

| **Strains or plasmids** | **Characteristics** | **Source** |
| --- | --- | --- |
| **Strains** | | |
| *Acremonium chrysogenum* 3.3795 | Wild-type strain | ^a^CGMCC |
| WT/pAg1PT-G418 | Negative control strain | This study |
| AcsamsOE | The *AcsamS* overexpressed strain | This study |
| Acppm1DM | The *Acppm1* disruption mutant | This study |
| Acppm1CM | The complemented strain of Acppm1DM | This study |
| Acppm1OE | The *Acppm1* overexpressed strain | This study |
| Acppm1DM-mecBOE | The *mecB* overexpressed strain based on Acppm1DM | This study |
| Acppm1DM-mecBOE-AcsamsOE | The *AcsamS* overexpressed strain based on Acppm1DM-mecBOE | This study |
| *Escherichia coli* DH5α | Used for routine cloning | Gibco BRL |
| *Bacillus subtilis* 1.1630 | Indicator strain for detection of cephalosporin production | ^a^CGMCC |
| *Agrobacterium tumefaciens* | Used for ATMT | [1] |
| **Plasmids** | | |
| pEASY-Blunt | Routine cloning vector | Transgen |
| pEBPgpd | DNA fragment containing the promoter region of *Acgapdh* was inserted into pEASY-Blunt | This study |
| pEBTgpd | DNA fragment containing the terminator region of *Acgapdh* was inserted into pEASY-Blunt | This study |
| pAg1-H3 | The vector used for ATMT | [1] |
| pAgHG | The G418 resistant gene was inserted into pAg1-H3 | [2] |
| pAg1PT-G418 | The promoter region and terminator region of *gapdh* was inserted into pAgHG | This study |
| pEBAcsamsOE | cDNA of *AcsamS* was inserted into pEASY-Blunt | This study |
| pAg1PT-G418::AcsamS | cDNA of *AcsamS* was inserted into pAg1PT-G418 | This study |
| pAg1PT-G418-AcsamS-ble | The bleomycin resistant gene (*ble*) was inserted into pAg1PT-G418-AcsamS | This study |
| pEBppm1LB | DNA fragment containing the upstream flanking sequence of *Acppm1* was inserted into pEASY-Blunt | This study |
| pEBppm1RB | DNA fragment containing the downstream flanking sequence of *Acppm1* was inserted into pEASY-Blunt | This study |
| pAgHB | The bleomycin resistant gene (*ble*) was insert into pAg1-H3 | [3] |
| pAgHBppm1LB | DNA fragment containing the upstream flanking sequence of *Acppm1* was inserted into pAgHB | This study |
| pAgHBppm1LR | DNA fragment containing the downstream flanking sequence of *Acppm1* was inserted into pAgHBppm1LB | This study |
| pEBppm1C | DNA of *Acppm1* with its promoter and terminator was inserted into pEASY-Blunt | This study |
| pAgB | The hygromycin phosphotransferase gene (*hph*) was deleted from pAgHB | [3] |
| pAgB::Acppm1C | DNA fragment containing *Acppm1* with its promoter and terminator was inserted into pAgB | This study |
| pEBppm1OE | cDNA of *Acppm1* was inserted into pEASY-Blunt | This study |
| pAgH-Pgpd | The promoter of *gapdh* was inserted into pAg1-H3 | [3] |
| pAgHP::Acppm1 | cDNA of *Acppm1* was inserted into pAgH-Pgpd | This study |
| pEBmecBOE | cDNA of *mecB* was inserted into pEASY-Blunt | This study |
| pAg1PT-G418::mecB | cDNA of *mecB* was inserted into pAg1PT-G418 | This study |
| pJL43-RNAi | Used for providing the bleomycin resistant gene (*ble*) together with promoter and terminator | [4] |

^a^CGMCC indicates China General Microbiological Culture Collection Center.

**Table S2 Primers used in this study.**

| **Primers** | **Sequence (5’-3’)** |
| --- | --- |
| Pgpd-F | gtcgacCCAGCGTAGAAGGCAGAGGT |
| Pgpd-R | gtcgacTTTGATTGATCTGGGAAGATTATTAG |
| Tgpd-F | actagtGTATGTTGTCGTCGGGAAGCC |
| Tgpd-R | actagtTGGTAAAAACGAGCAAGCGAG |
| G418-F | agatctTTAACGCTTACAATTTCC |
| G418-R | agatctGAATAGGAACTTCGGAAT |
| ble-F  ble-R | TTGCGACGGCGTATTGCTTA  CCTCGAGGAATTCGATATCA |
| Acppm1LB-F | GGTCGTCGTCGGTGTTGA |
| Acppm1LB-R | CGAGCGTGAGGAGTTCTTG |
| Acppm1RB-F | CCCGTAGCAAATGTATTGTATG |
| Acppm1RB-R | TGAGGGTTGGTTGTGGTGT |
| Acppm1Out-F | CCTCCGTGGATAGTCTCCGT |
| Acppm1Out-R | CAACAAGCGGTCAAGTTGGTTC |
| Acppm1Int-F | AAGTCCCTTCAACAGTCGGC |
| Acppm1Int-R | CCACGGGTCACTCACATCC |
| Sppm1-F | GGGGGTTGGTGTCTATGTCA |
| Sppm1-R | CGCTCAATCAAGACCAGTTTC |
| Acppm1C-F | GGGAAGCGGAAGATGAGAC |
| Acppm1C-F | GAGAACAGCAGGATGAAGACG |
| AcsmasOE-F | ttaattaaATGTCTGCCAACGGCATCAAGGGCGT |
| AcsamsOE-R | ggcgcgccTTAGAACTTGAGAGGCTTGGGCTGCTCC |
| Acppm1OE-F | cccgggCACACCATCGTCGTTCGAGT |
| Acppm1OE-R | cccgggCGGAGAATCCAAAGAGCGAC |
| mecBOE-F | ttaattaaATGTCTCCTACAGCGGCACC |
| mecBOE-R | ggcgcgccTTATTGGCCCTTGACACCATT |
| RT-actin-F | AGTCCAAGCGTGGTATCC |
| RT-actin-R | TAGAAGGCAGGGGCGTTG |
| RT-Acsams-F | CTATGCCACCGACGAGACTC |
| RT-Acsams-R | TGGGCGGAGATGACAACA |
| RT-mecB-F | ATCGCCCTCAAGCAGCAC |
| RT-mecB-R | TCAACACCGCAGCTCACG |
| RT-Acppm1-F/RT-W-F | TGGACTTTGATGTCGTCTGCAG |
| RT-Acppm1-R/ RT-W-R | CTGCTTCCGTGTCTATGGGTTT |
| RT-cysD-F  RT-cysD-R  RT-mecA-F  RT-mecA-R  RT-metH-F  RT-metH-R | ATGTCGGACCTCAGCAAGAAC  GATGCGGCTGTAGATGTTGC  CGTCTTTCCGAGAAGCACAAC  GTGGGTCGAGGTGAATAGGG  AGGTCGCTGACTGGTTCTCC  AACTCGGACTTGCGCTTGTAG |
| RT-A-F | GATGGAACTCGCCTTTGTCAG |
| RT-A-R | AACTTCAGCCGCCTCTCTCC |
| RT-B-F | CGCCGACTTTGTCATCAGC |
| RT-B-R | ACGAGCGTGTCCTGGTCG |
| RT-C-F | GACCAGCAGAGTGAATCGCA |
| RT-C-R | GATGTCTGTTCCGATGACCTTG |
| RT-D-F | TGGCACGGGTGTCGGTTA |
| RT-D-R | TGCGTCCTCGTAGATGTTGGT |
| RT-E-F | GGGAGTCGTGGCAGCAGTAC |
| RT-E-R | CAAGTAGCGGCGTCCAGG |
| RT-F-F | ACGGATGGATATGACCCTGTTC |
| RT-F-R | TTCCTCTAGCTGCTGCTCTGG |
| RT-G-F | TGTCTACGCTTCGACCAACG |
| RT-G-R | CGTCCAGGATCTCCGTGCTA |
| RT-H-F | TTGTCCGACTGAGCGTTGAAA |
| RT-H-R | TGAGTGGAGTTTCTTTGGGGC |
| RT-I-F | TCGACTCTTGCTCAGCCAAA |
| RT-I-R | GTTGGTGCTGAGCCTCTTCTT |
| RT-J-F | CCCTGAGCGAGCACACCAT |
| RT-J-R | ACGATTCCGCAGCCAGGTA |
| RT-K-F | GCTTCTTCCACCTCTTCACCTG |
| RT-K-R | ATTCTCCCAACCTCGTTCCAG |
| RT-L-F | CCGGAACACAATCCTACCCC |
| RT-L-R | TTTGGAGCGGTTACTGGCAC |
| RT-M-F | AGCTTGCCAAACGTGATGC |
| RT-M-R | CCTGCCTCAGCCGTCTTCT |
| RT-N-F | GCTCTTGCCGCTCACCTCAC |
| RT-N-R | GTCGTCACCAAGGACGGAAAGT |
| RT-O-F | GGACGCCGATCAAGGTCTATG |
| RT-O-R | CGCATATTCTTCTCCGGCTCT |
| RT-P-F | TCTACGGCGTGGAACCAAAC |
| RT-P-R | GGCACAGGATGGAGACGATG |
| RT-Q-F | CCTCGTCGGGGAAGAACCA |
| RT-Q-R | CCACTGGGTCCTCCGCTGT |
| RT-R-F | CAACCAGATTGCCTCGGACT |
| RT-R-R | CTGTCGGAGATGTCTATCCCG |
| RT-S-F | GCCCTAGCGAGGAAGAGTTGT |
| RT-S-R | GCACCAGCCGACAGTCTTATTA |
| RT-T-F | AAGGGTGCGTTGTGGGATAG |
| RT-T-R | GTCCACGCAATAGGCGAAAT |
| RT-U-F | GCCGTCTGTCAGTTCTACCCC |
| RT-U-R | ACACCAGTGATGTCGCCCC |
| RT-V-F | AGGGTGCGTCAAGGAGAGGT |
| RT-V-R | CACTTCTTCCGAGCAAGTACCAT |

**
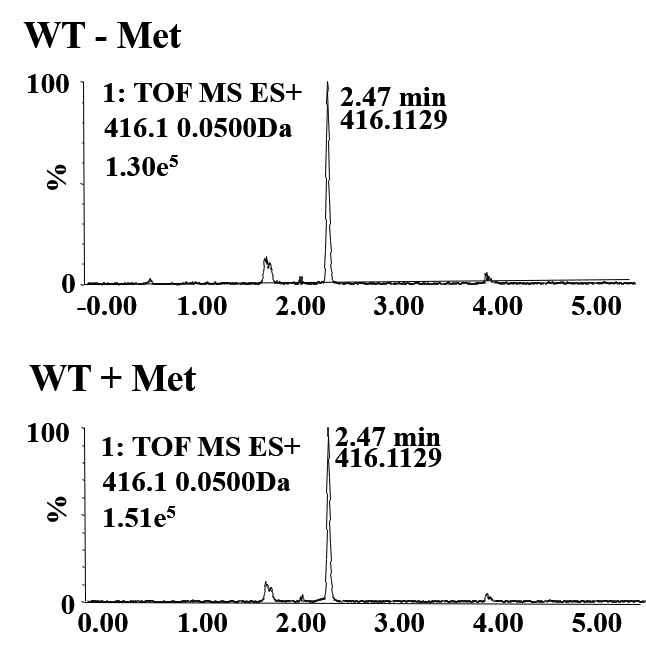
**

**Fig. S1** Cephalosporin C production of WT detected by UPLC/MS in the MDFA medium with or without addition of 3.2 g/L methionine. CPC production was detected by UPLC/MS as described in Methods. The quantitative signal intensity of CPC from the fermentation without methionine is 1.30e5, the quantitative signal intensity of CPC from the fermentation with methionine is 1.51e5.


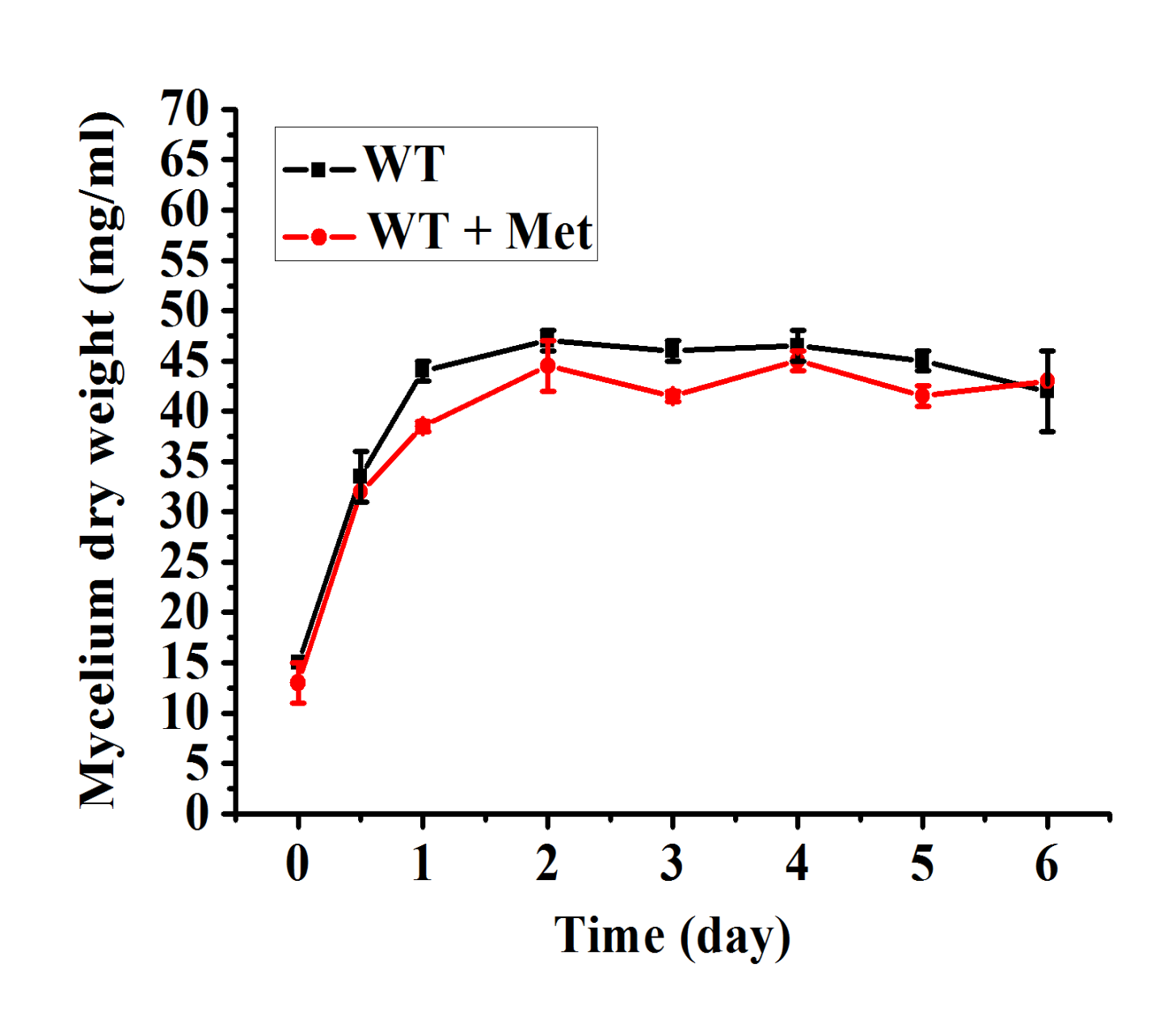


**Fig. S2** Mycelium dry weight of *A. chrysogenum* in the MDFA medium with or without addition of 3.2 g/L methionine. Mycelium dry weight of the *A. chrysogenum* wild-type strain (WT) was determined after drying the fungal mycelia at 42 ^o^C in a hot air oven until a constant weight. Met, methionine. Error bars represent standard deviations from three independent experiments.

**
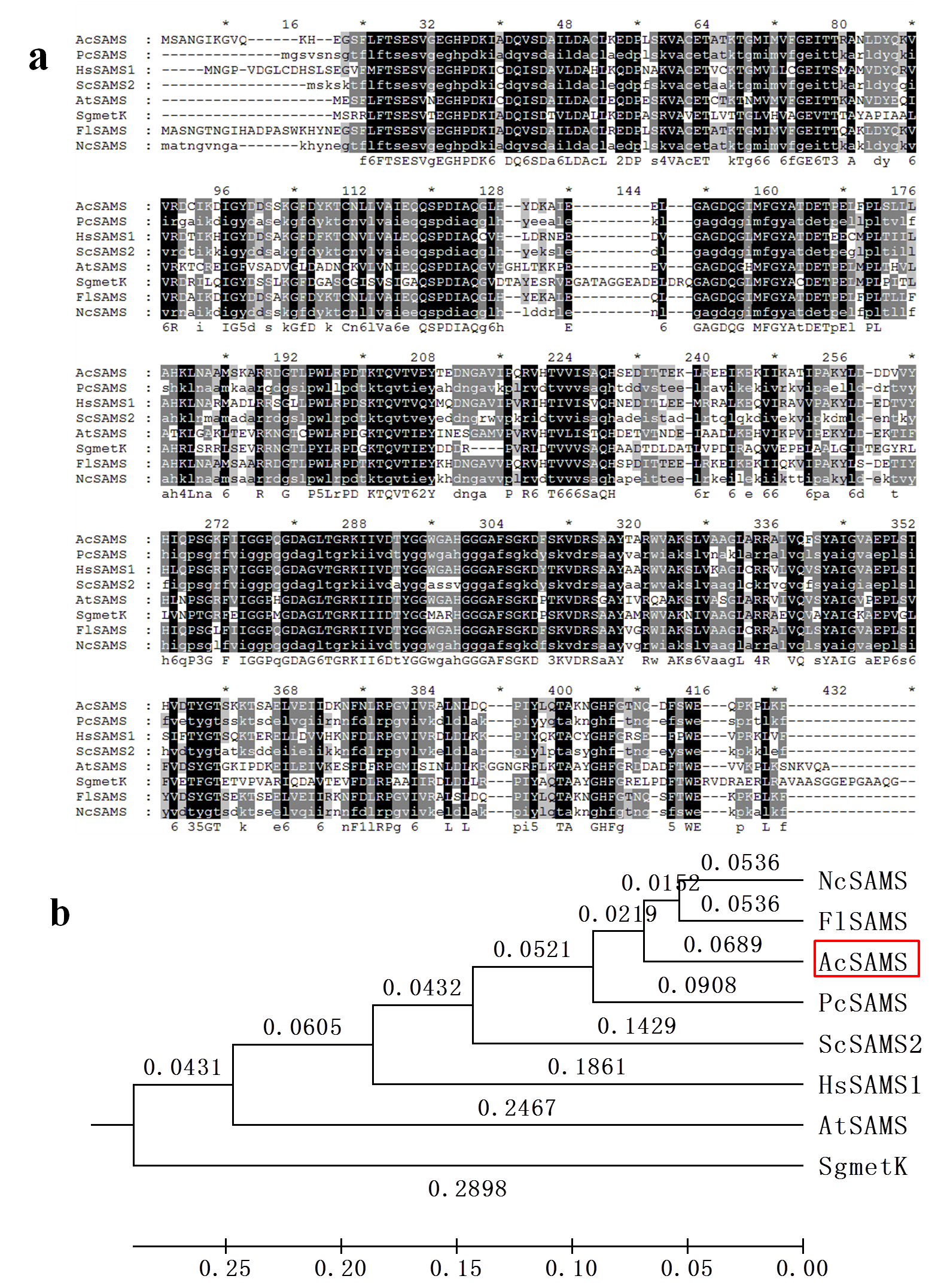
**

**Fig. S3** Sequence alignment and phylogenetic analysis of the SAM synthetase family proteins. (a) Sequence alignment of AcSAMS (GenBank accession No. MG356328) from *A. chrysogenum*, PcSAMS from *P. chrysogenum*, ScSAMS2 from *S. cerevisiae*, HsSAMS1 from *Homo*, AtSAMS from *A. thaliana*, SgmetK from *S. griseofuscus*, FlSAMS from *F. langsethiae* and NcSAMS from *N. crassa*. (b) Phylogenetic analysis of AcSAMS and its orthologs. The related sequences were collected from NCBI database. The phylogenetic tree was constructed using the neighbor-joining method (p-distance model) with MEGA 5 software.


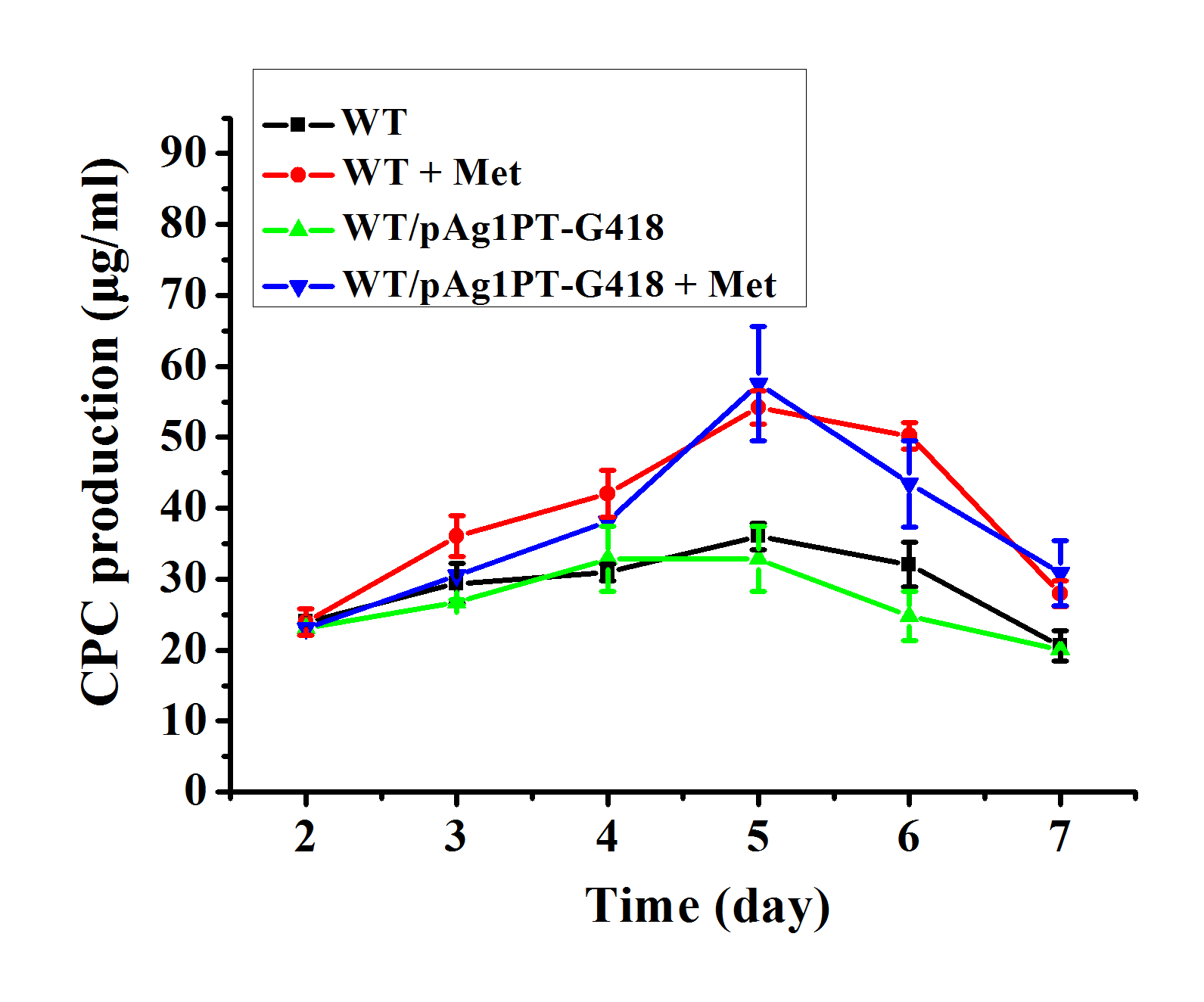


**Fig. S4** Cephalosporin C production of WT and WT/pAg1PT-G418 in the MDFA medium with or without addition of 3.2g/L methionine. CPC production was determined by bioassays against *B. subtilis* 1.1630 as described in Methods. WT, the wild-type strain; WT/pAg1PT-G418, the wild-type strain carrying the plasmid pAg1PT-G418. The fermentation was performed in the MDFA medium with or without addition of 3.2g/L methionine. Error bars represent standard deviations from three independent experiments.


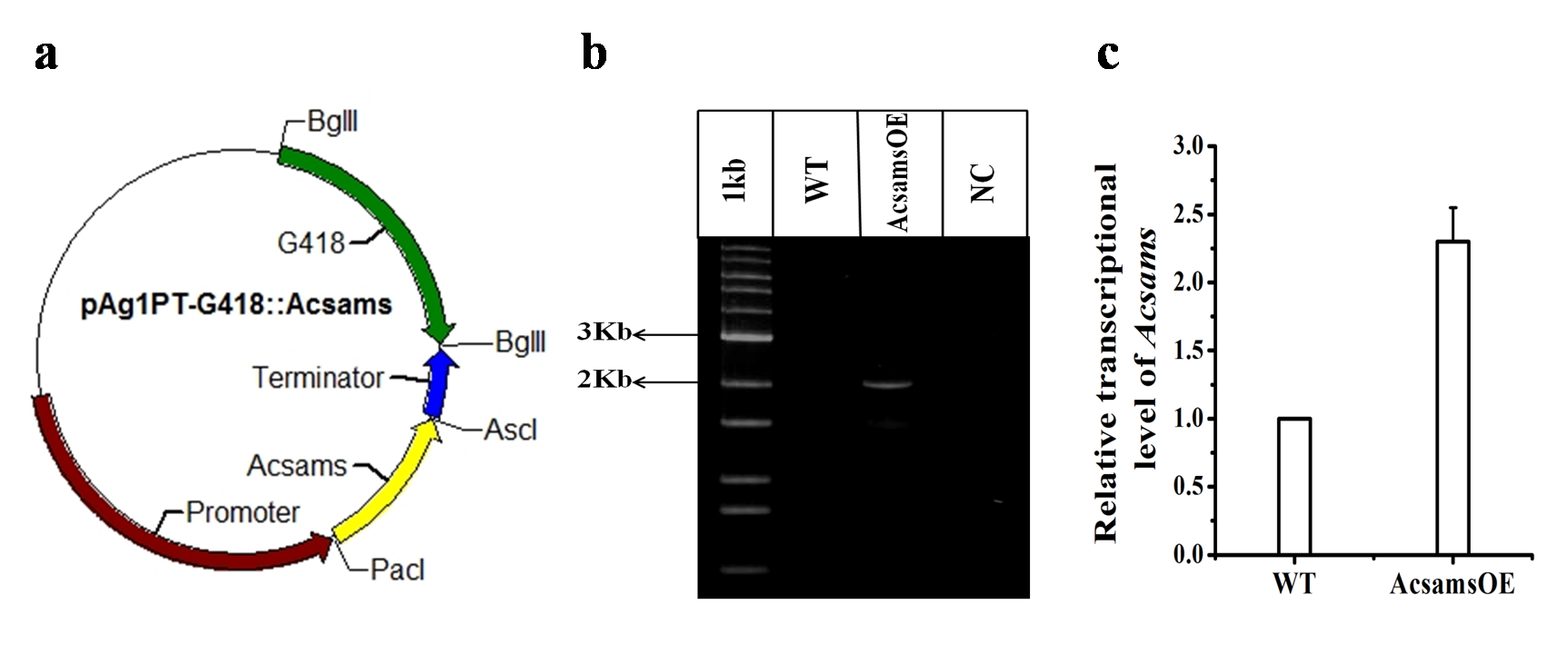


**Fig. S5** Construction and validation of the *AcsamS* overexpressed strain (AcsamsOE). (a) The plasmid pAg1PT-G418::AcsamS was used for overexpressing *AcsamS*. Promoter, the promoter of glyceraldehyde-3-phosphate dehydrogenase (GAPDH) gene; Terminator, the terminator of glyceraldehyde-3-phosphate dehydrogenase (GAPDH) gene. (b) Validation of AcsamsOE by PCR. The G418 resistant gene (*G418*) was amplified by PCR with primers G418-F/G418R. WT, the wild-type strain of *A. chrysogenum*; AcsamsOE, the *AcsamS* overexpressed strain; NC, negative control. (c) Relative transcriptional level of *AcsamS* in WT and AcsamsOE. Both strains were grown in TSA liquid medium at 28 °C for 2 days. Total RNA extraction and cDNA synthesis were performed as described in Methods. The relative transcriptional level of *AcsamS* was detected by real-time RT-PCR with primers RT-Acsams-F/R. The relative abundance of mRNAs was standardized against to the level of *actin* gene. Error bars represent standard deviations from three independent experiments.


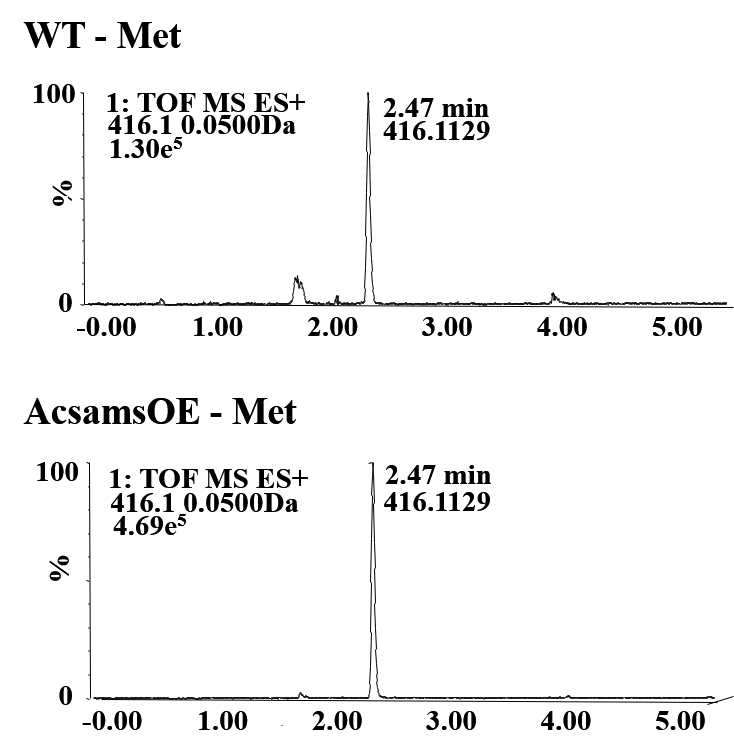


**Fig. S6** Cephalosporin C production of WT and AcsamsOE was detected by UPLC/MS in MDFA medium. CPC production of WT and AcsamsOE in the MDFA medium without addition of methionine was determined with UPLC/MS as described in Methods. The quantitative signal intensity of CPC from WT is 1.30e5, the quantitative signal intensity of CPC from AcsamsOE is 4.69e5.


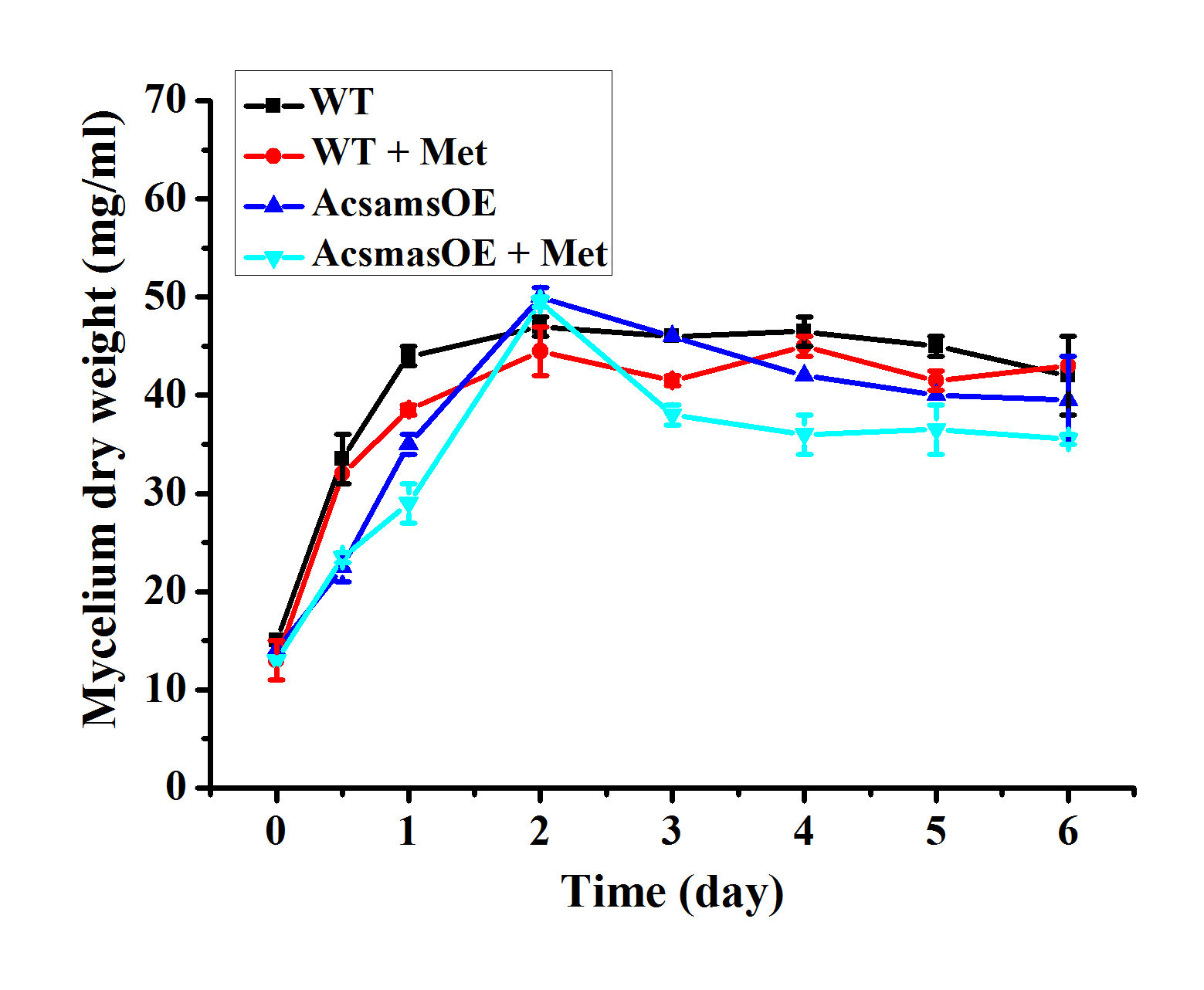


**Fig. S7** Mycelium dry weight of AcsamsOE in the MDFA medium with or without addition of 3.2 g/L methionine. Mycelium dry weight was determined after drying the fungal mycelia at 42 ^o^C in a hot air oven until a constant weight. WT, the *A. chrysogenum* wild-type strain; AcsamsOE, the *AcsamS* overexpressed strain; Met, methionine. Error bars represent standard deviations from three independent experiments.


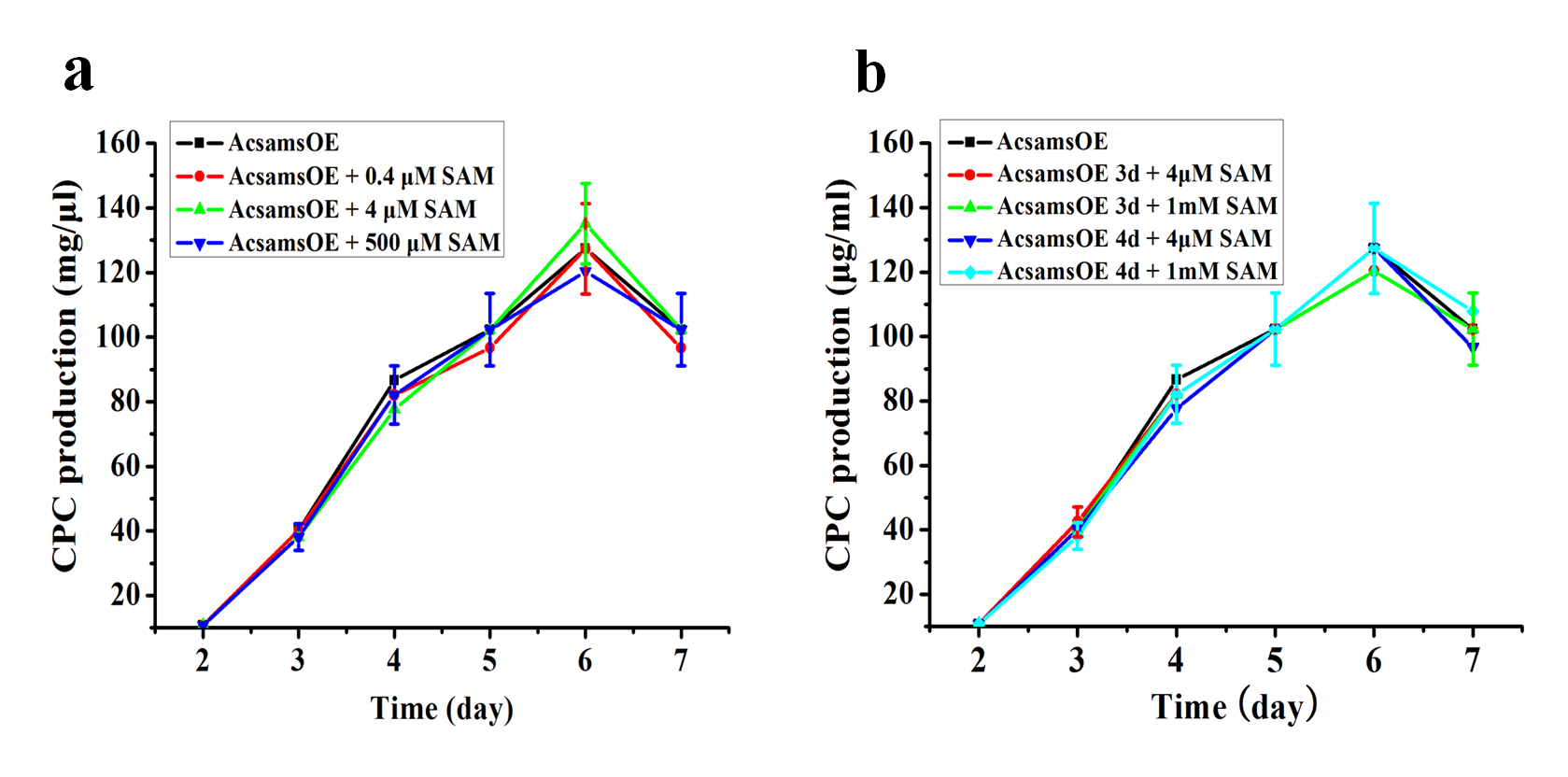


**Fig. S8** Cephalosporin C production of AcsamsOE in the MDFA medium supplemented with different concentration of SAM. (a) CPC production of AcsamsOE in the MDFA medium supplemented with 0, 0.4 μM, 4 μM and 500 μM of SAM. (b) CPC production of AcsamsOE in the MDFA medium supplemented with 4 μM and 1 mM SAM respectively after 3 or 4 days fermentation. CPC production was determined by bioassays against *B. subtilis* 1.1630 as described in Methods. Error bars represent standard deviations from three independent experiments.


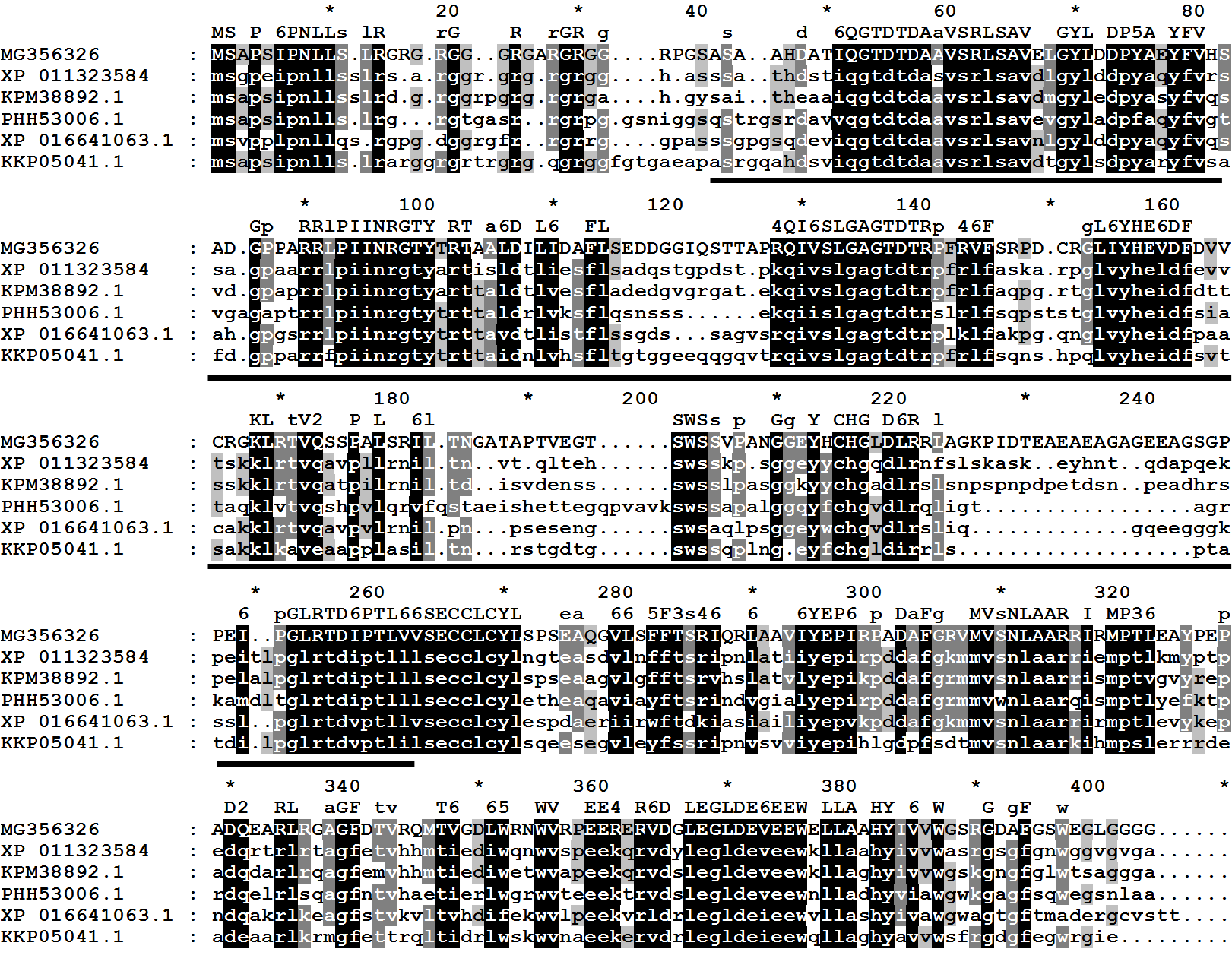


**Fig. S9** Sequence alignment of the leucine carboxyl methyltransferase superfamily proteins. AcPPM1 (GenBank accession No. MG356326) from *A. chrysogenum*, XP_011323584.1 from *F. graminearum*, KPM38892.1 from *N. ditissima*, PHH53006.1 from *C. fimbriata*, XP_016641023.1 from *S. apiospermum*, KKP05041.1 from *T. harzianum*. The conserved SAM dependent methyltransferase domain was underlined.


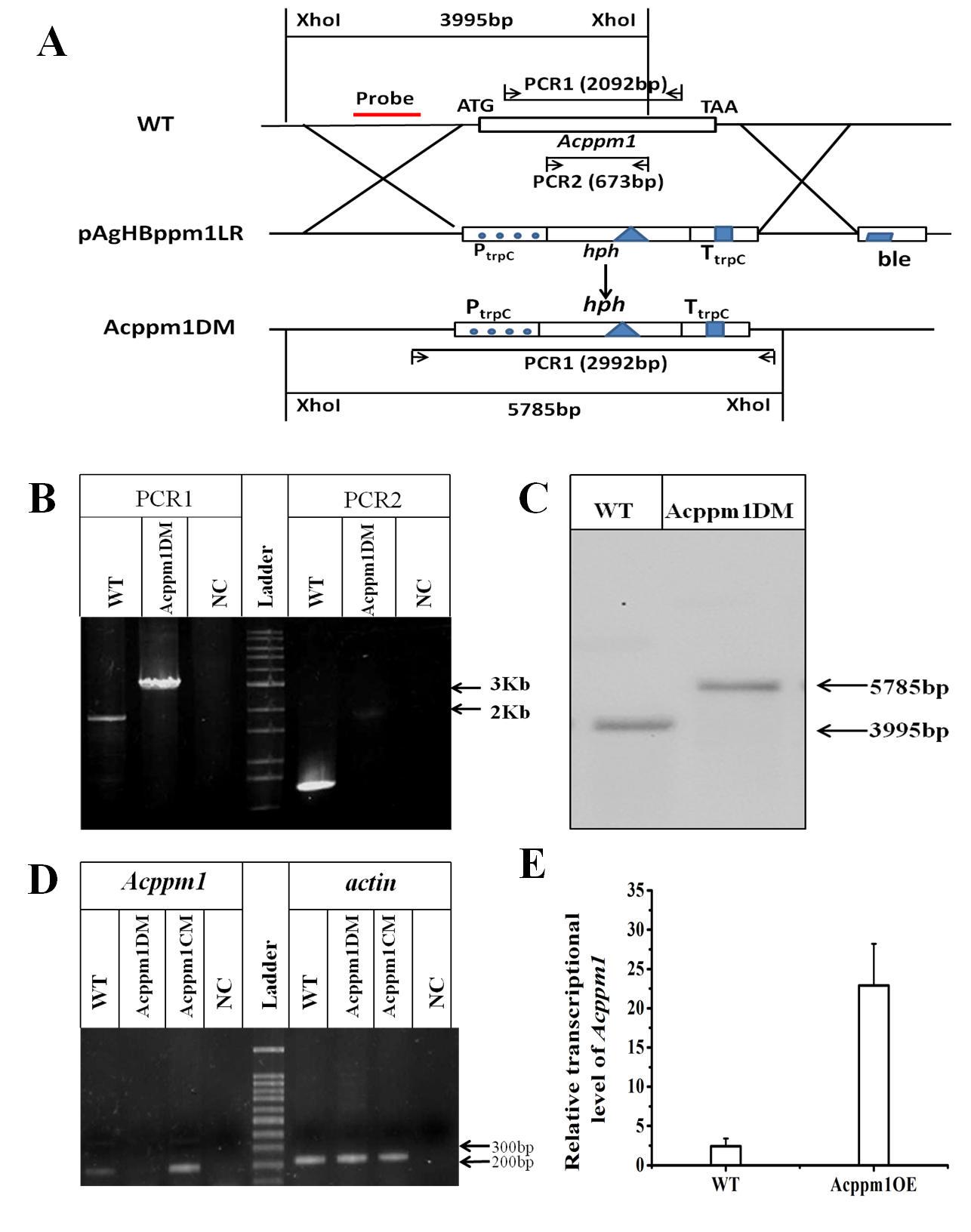


**Fig. S10** Construction and validation of the *Acppm1* disruption mutant (Acppm1DM). (a) Strategy for construction of Acppm1DM via homologous recombination. bp, base pairs; *hph*, the hygromycin B phosphotransferase gene; *ble*, the bleomycin resistant gene; Probe, the probe used for Southern hybridization. (b) Validation of Acppm1DM by PCR. PCR1 and PCR2 were performed with primers Acppm1Out-F/R and Acppm1Int-F/R, respectively. Ladder, 1 kb ladder; WT, the wild-type strain; NC, negative control. (c) Validation of Acppm1DM by Southern hybridization. A 735 bp DNA fragment next to *Acppm1* is labeled by digoxigenin-11-dUTP and used as probe. The genome DNAs from WT and Acppm1DM were digested with *Xho*I. (d) Transcriptional analysis of *Acppm1* in WT, Acppm1DM and the complemented strain (Acppm1CM). Total RNA extraction and cDNA synthesis were performed as described in Methods. Transcription of *Acppm1* was detected by semi-quantitation RT-PCR with primers RT-Acppm1-F/R, the transcription of *actin* gene was used as control. WT, the wild-type strain; Acppm1DM, the *Acppm1* disruption mutant; Acppm1CM, the complemented strain; NC, negative control. (e) The relative transcriptional level of *Acppm1* in WT and the *Acppm1* overexpressed strain (Acppm1OE). Both strains were grown in TSA liquid medium at 28 °C for 2 days. Total RNA extraction and cDNA synthesis were performed as described in Methods. The relative transcriptional level of *Acppm1* was detected by real-time RT-PCR with primers RT-Acppm1-F/R. The relative abundance of mRNAs was standardized against to the level of *actin* gene. Error bars represent standard deviations from three independent experiments.


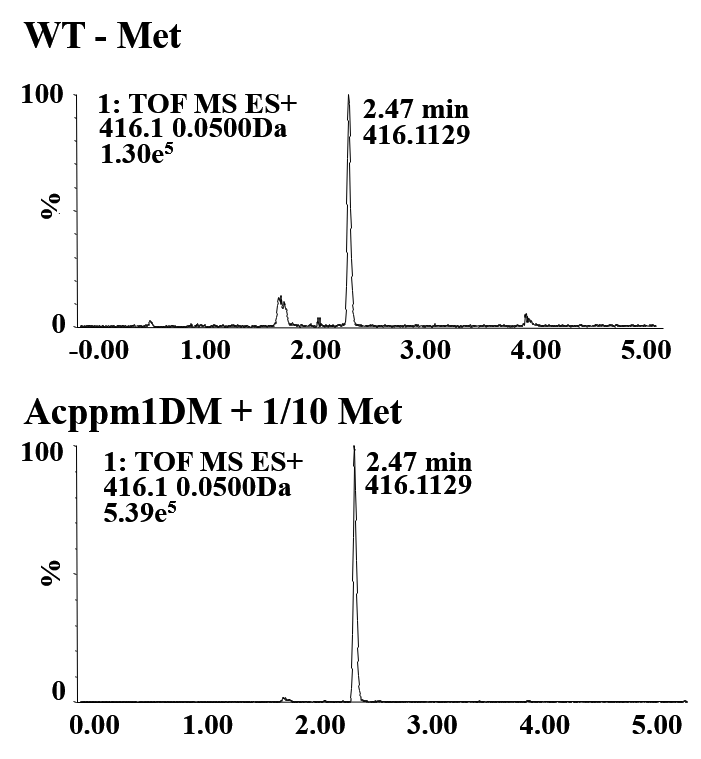


**Fig. S11** Cephalosporin C production of WT and Acppm1DM was detected by UPLC/MS. CPC production was determined with UPLC/MS as described in Methods. The quantitative signal intensity of CPC from WT is 1.30e5, the quantitative signal intensity of CPC from Acppm1DM is 5.39e5.


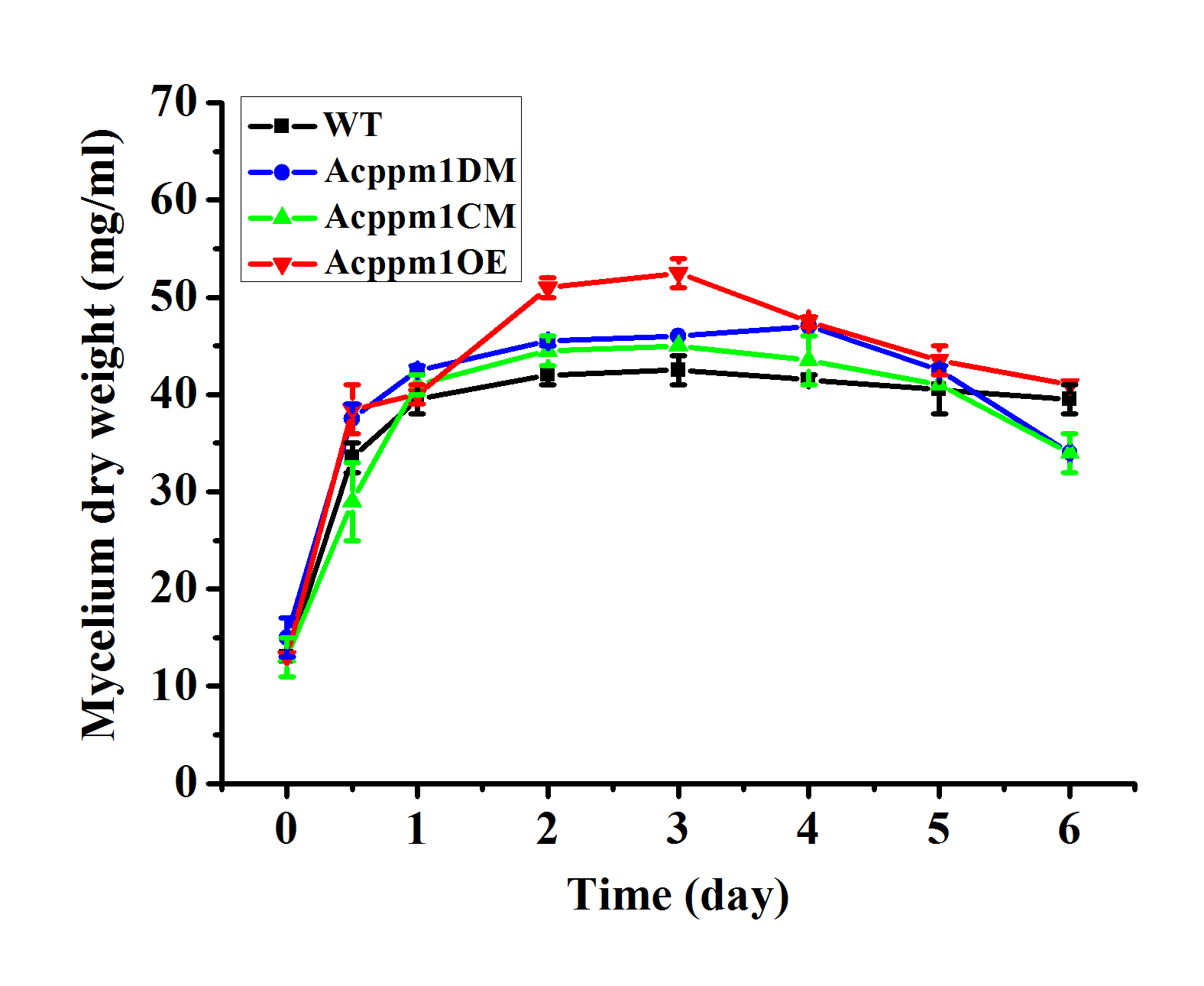


**Fig. S12** Mycelium dry weight of Acppm1DM, Acppm1CM, Acppm1OE in the MDFA medium with or without addition of 0.32 g/L methionine. Mycelium dry weight was determined after drying the fungal mycelia at 42 ^o^C in a hot air oven until a constant weight. The external addition of methionine was 0.32 g/L. WT, the wild-type strain; Acppm1DM, the *Acppm1* disruption mutant; Acppm1CM, the complemented strain of Acppm1DM with a copy of *Acppm1*; Acppm1OE, the *Acppm1* overexpressed strain. Error bars represent standard deviations from three independent experiments.


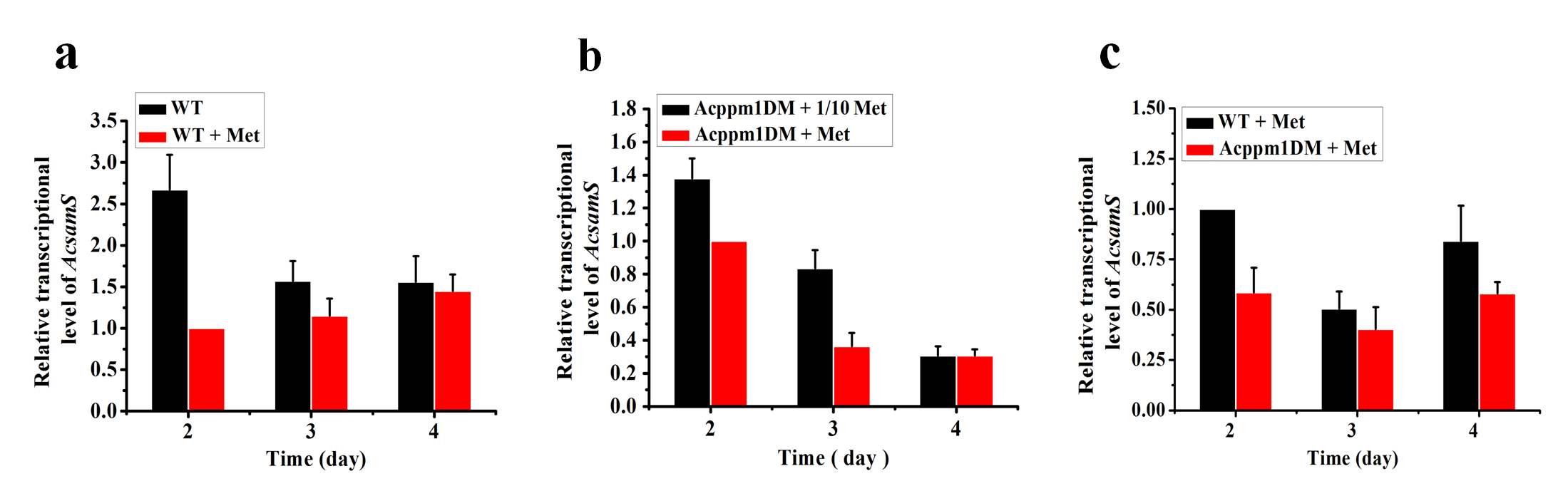


**Fig. S13** The relative transcriptional level of *AcsamS* in WT and Acppm1DM. Total RNA extraction and cDNA synthesis were performed as described in Methods. The relative transcriptional level of *AcsamS* was detected by real-time RT-PCR in WT and Acppm1DM which were grown in MDFA supplemented with 0, 0.32 g/L or 3.2 g/L methionine respectively. The relative abundance of mRNAs was standardized against to the level of *actin* gene. Error bars represent standard deviations from three independent experiments.


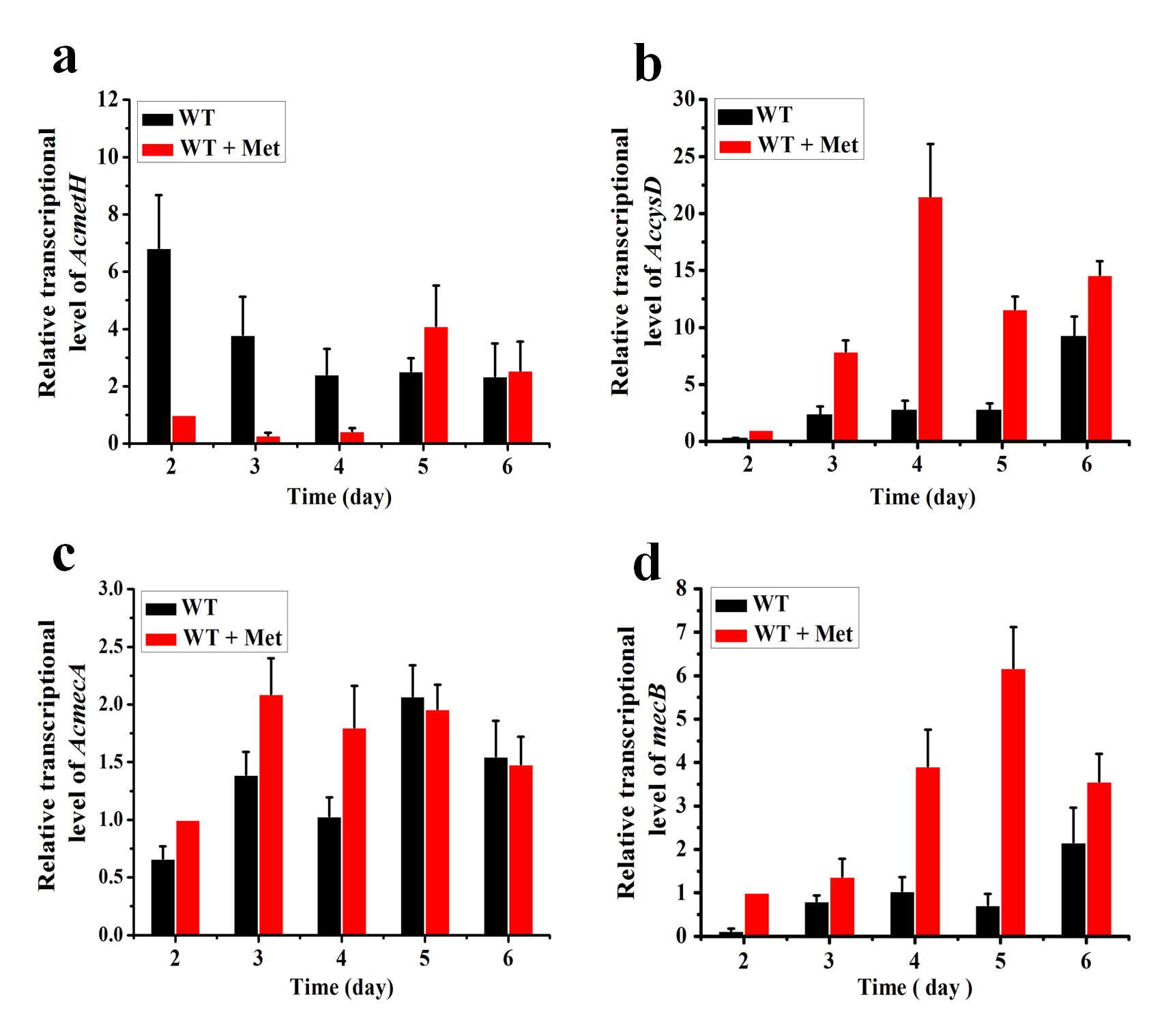


**Fig. S14** The relative transcriptional level of *AcmetH*, *AccysD*, *AcmecA and mecB* of WT in the MDFA medium with or without addition of 3.2 g/L methionine. Total RNA extraction and cDNA synthesis were performed as described in Methods. The relative transcriptional level of *AcmetH*, *AccysD*, *AcmecA* and *mecB* was detected by real-time RT-PCR using primers RT-AcmetH-F/R, RT-AccysD-F/R, RT-AcmecA-F/R and RT-mecB-F/R. The relative abundance of mRNAs was standardized against to the level of *actin* gene. Error bars represent standard deviations from three independent experiments.


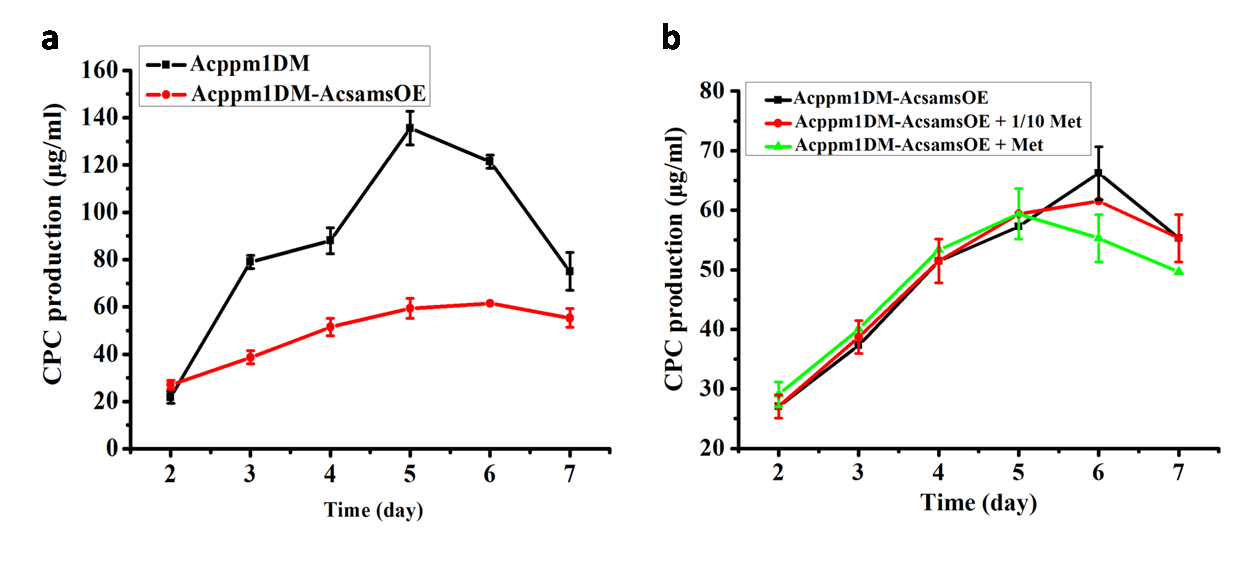


**Fig. S15** Cephalosporin C production of Acppm1DM and Acppm1DM-AcsamsOE. (a) CPC production of Acppm1DM and Acppm1DM-AcsamsOE in the MDFA medium supplemented with 0.32 g/L methionine. (b) CPC production of Acppm1DM-AcsamsOE in the MDFA medium supplemented with 0, 0.32 g/L and 3.2 g/L methionine respectively. CPC production was determined by bioassays against *B. subtilis* 1.1630 as described in Methods. Error bars represent standard deviations from three independent experiments.


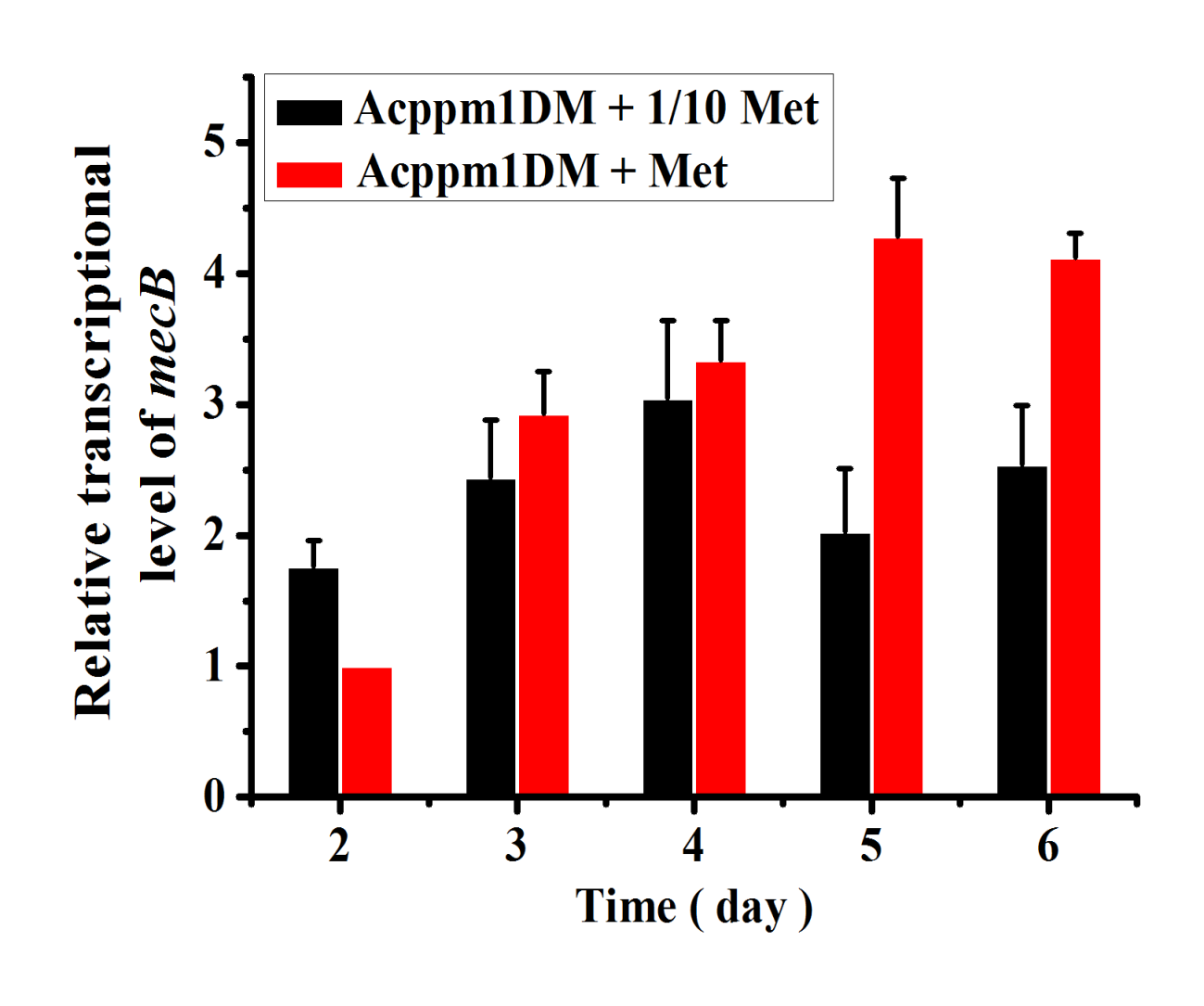


**Fig. S16** The relative transcriptional level of *mecB* in Acppm1DM. Acppm1DM was grown in the MDFA medium supplemented with 0.32 g/L and 3.2 g/L of methionine respectively. Acppm1DM+1/10 Met, the *Acppm1* disruption mutant grown in the MDFA medium with addition of 0.32 g/L of methionine; Acppm1DM+Met, the *Acppm1* disruption mutant grown in the MDFA medium with addition of 3.2 g/L of methionine. Total RNA extraction and cDNA synthesis were performed as described in Methods. The relative transcriptional level of *mecB* was detected by real-time RT-PCR. The relative abundance of mRNAs was standardized against to the level of *actin* gene. Error bars represent standard deviations from three independent experiments.


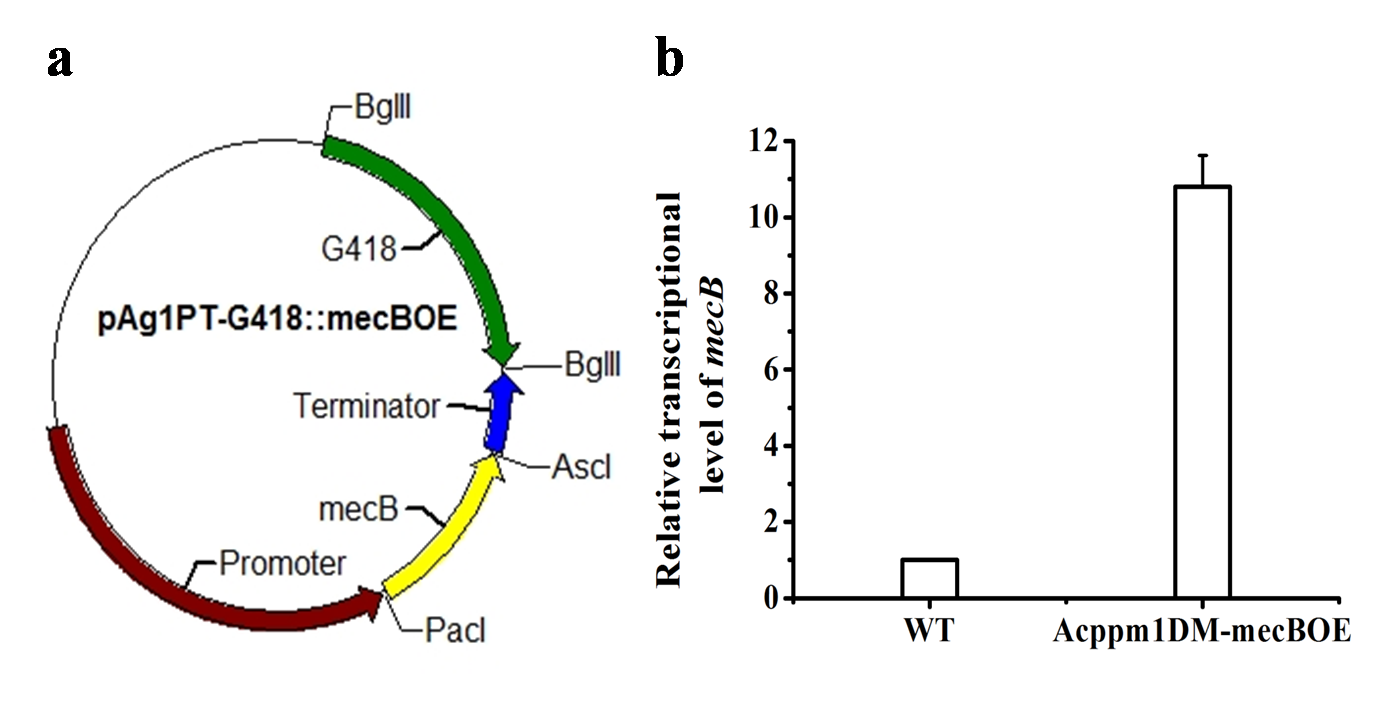


**Fig. S17** Construction and validation of the *mecB* overexpressed strain (Acppm1DM-mecBOE). (A) The plasmid pAg1PT-G418::mecB used for *mecB* overexpression. Promoter, the promoter of glyceraldehyde-3-phosphate dehydrogenase (GAPDH) gene; Terminator, the terminator of glyceraldehyde-3-phosphate dehydrogenase (GAPDH) gene. (B) The relative transcriptional level of *mecB* in WT and Acppm1DM. Both strains were grown in TSA liquid medium at 28 °C for 2 days. Total RNA extraction and cDNA synthesis were performed as described in Methods. The relative transcriptional level of *mecB* was detected by real-time RT-PCR with primers RT-mecB-F/R. The relative abundance of mRNAs was standardized against to the level of *actin* gene. WT, the wild-type strain; Acppm1DM-mecBOE, the *mecB* was overexpressed in Acppm1DM. Error bars represent standard deviations from three independent experiments.


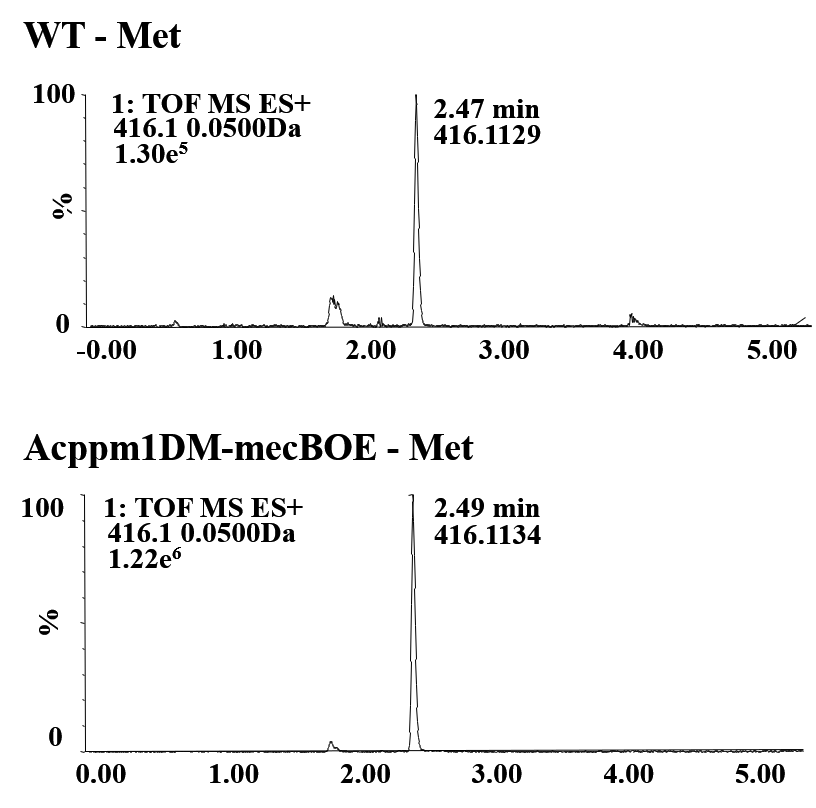


**Fig. S18** Cephalosporin C production of WT and Acppm1DM-mecBOE was detected by UPLC/MS in MDFA medium. CPC production of WT and Acppm1DM-mecBOE in the MDFA medium without addition of methionine was determined with UPLC/MS as described in Methods. The quantitative signal intensity of CPC from WT is 1.30e5, the quantitative signal intensity of CPC from Acppm1DM-mecBOE is 1.22e6.


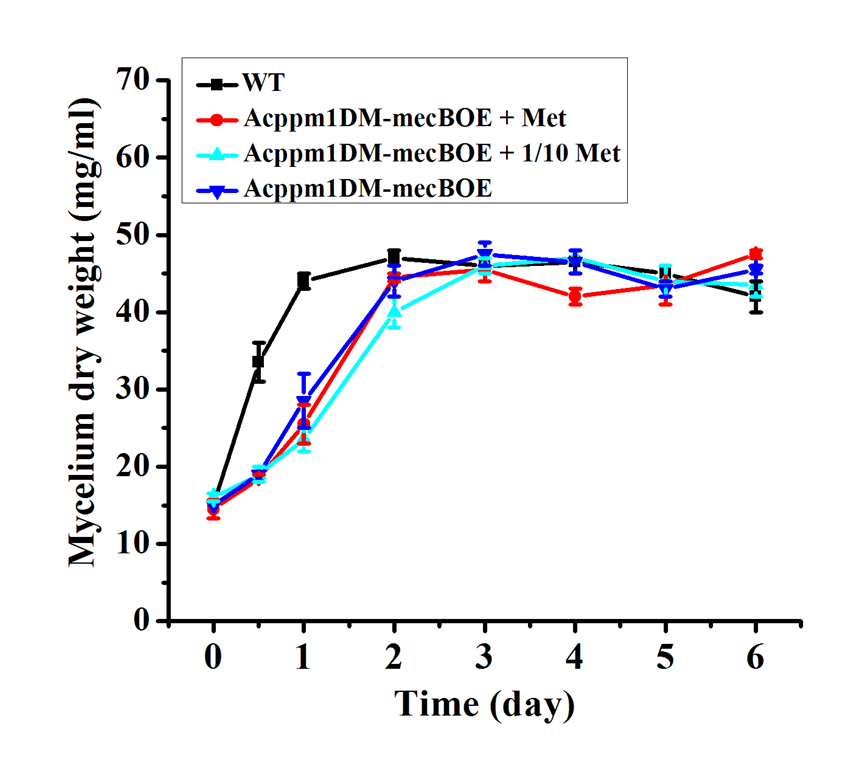


**Fig. S19** Mycelium dry weight of WT and Acppm1DM-mecBOE in the MDFA medium supplemented with 0, 0.32 g/L and 3.2 g/L of methionine. Mycelium dry weight was determined after drying the fungal mycelia at 42 ^o^C in a hot air oven until a constant weight. +Met, 3.2 g/L of methionine was added in MDFA medium; + 1/10 Met, 0.32 g/L of methionine was added in MDFA medium. Error bars represent standard deviations from three independent experiments.


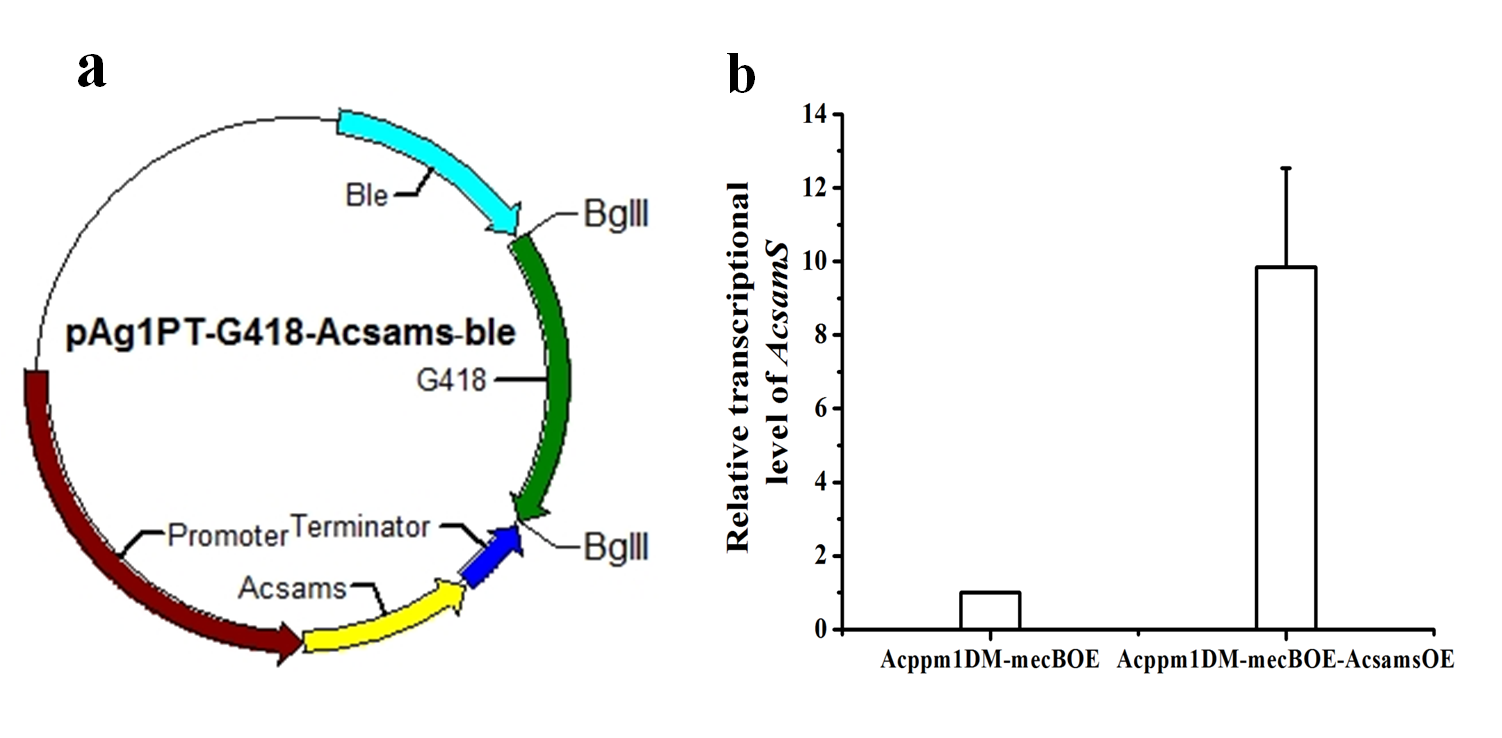


**Fig. S20** Construction and validation of Acppm1DM-mecBOE-AcsamsOE. (A) The plasmid pAg1PT-G418-AcsamS-ble was used for *AcsamS* overexpression. Promoter, the promoter of glyceraldehyde-3-phosphate dehydrogenase (GAPDH) gene; Terminator, the terminator of glyceraldehyde-3-phosphate dehydrogenase (GAPDH) gene; *ble*, the fragment contained the entire bleomycin resistant gene (*ble*) together with its promoter and terminator. (B) The relative transcriptional level of *AcsamS* in Acppm1DM-mecBOE and Acppm1DM-mecBOE-AcsamsOE. Acppm1DM-mecBOE, *mecB* was overexpressed in Acppm1DM; Acppm1DM-mecBOE-AcsamsOE, *AcsamS* was overexpressed in Acppm1DM-mecBOE. Both strains were grown in TSA liquid medium at 28 °C for 2 days. Total RNA extraction and cDNA synthesis were performed as described in Methods. The relative transcriptional level of *AcsamS* was detected by real-time RT-PCR with primers RT-Acsams-F/R. The relative abundance of mRNAs was standardized against to the level of *actin* gene. Error bars represent standard deviations from three independent experiments.


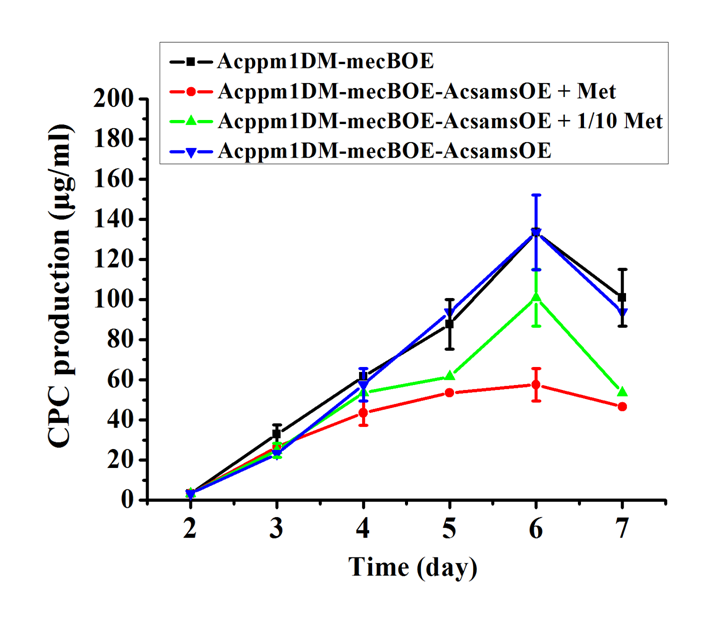


**Fig. S21** Cephalosporin C production of Acppm1DM-mecBOE-AcsamsOE. The CPC production of Acppm1DM-mecBOE-AcsamsOE was detected in the MDFA medium supplemented with 0, 0.32 g/L (1/10 of the normal level) and 3.2 g/L (normal level) methionine respectively. CPC production was determined by bioassays against *B. subtilis* 1.1630 as described in Methods. Error bars represent standard deviations from three independent experiments.

**Supplementary references**

1. Khang CH, Park SY, Rho HS, Lee YH, Kang S. Filamentous fungi (*Magnaporthe grisea* and *Fusarium oxysporum*). Methods Mol Biol. 2006; 344:403-420.
2. Wang Y, Hu P, Pan Y, Zhu Y, Liu X, Che Y, Liu G. Identification and characterization of the verticillin biosynthetic gene cluster in *Clonostachys rogersoniana*. Fungal Genet Biol. 2017; 103:25-33.
3. Li J, Pan Y, Liu G. Disruption of the nitrogen regulatory gene *AcareA* in *Acremonium chrysogenum* leads to reduction of cephalosporin production and repression of nitrogen metabolism. Fungal Genet Biol. 2013; 61:69-79.
4. Ullán RV, Godio RP, Teijeira F, Vaca I, García-Estrada C, Feltrer R, Kosalkova K, Martín JF. RNA-silencing in *Penicillium chrysogenum* and *Acremonium chrysogenum*: validation studies using beta-lactam genes expression. J Microbiol Methods. 2008;75:209-218.
